# Mechanical Forces Guide Axon Growth through the Nigrostriatal Pathway in an Organotypic Model

**DOI:** 10.1002/advs.202500400

**Published:** 2025-05-11

**Authors:** Sara De Vincentiis, Elena Capitanini, Karen Kira, Claudia Dell'Amico, Jun Takahashi, Marco Onorati, Fabian Raudzus, Vittoria Raffa

**Affiliations:** ^1^ Department of Biology University of Pisa Pisa 56126 Italy; ^2^ Department of Clinical Application Center for iPS Cell Research and Application (CiRA) Kyoto University Kyoto 606‐8507 Japan; ^3^ Neuronal Signaling and Regeneration Unit Graduate School of Medicine Kyoto University Kyoto 606‐8501 Japan; ^4^ Center for Medical Education and Internationalization (CMEI) Graduate School of Medicine Kyoto University Kyoto 606‐8501 Japan

**Keywords:** axon growth, cell replacement, dopaminergic progenitors, mechanical force, Parkinson, striatum, substantia nigra

## Abstract

Reconstructing the nigrostriatal pathway is one of the major challenges in cell replacement therapies for Parkinson's disease due to the lack of enabling technologies capable of guiding the reinnervation of dopaminergic precursors transplanted into the substantia nigra toward the striatum. This paper examines nano‐pulling, as a technology to enable the remote manipulation of axonal growth. Specifically, an organotypic model consisting of co‐cultures of the substantia nigra and the striatum is developed to demonstrate that when cortical neural progenitors are transplanted into the substantia nigra, nano‐pulling can guide and enhance the elongation of neural projections toward the striatum. To provide additional evidence, induced pluripotent stem cell‐derived dopaminergic progenitor neurospheres are generated and it is shown that nano‐pulling can induce guided growth and promote the maturation of their neural processes. Altogether, this study demonstrates the potential of nano‐pulling as an emerging technique to promote directed reinnervation within the central nervous system.

## Graphical abstract

Nano‐pulling is a magnetic nanoparticle‐based technology for guiding and promoting the axon growth of induced pluripotent stem cell‐derived dopaminergic neurospheres in vitro or neural progenitors transplanted in the brain tissue. Fine control of axon guidance is essential for reconstructing neural networks and restoring functional connectivity in the injured central nervous system.

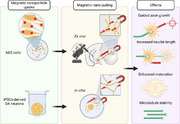

## 1 Introduction

Parkinson's disease (PD) is the second most frequent age‐related neurodegenerative disease globally. PD's prevalence has doubled over the past 25 years, with an alarming rise to 13 million patients projected by 2040.^[^ ^1^ ^]^ The progressive loss of dopaminergic (DAergic) neurons of the substantia nigra (SN) is considered the histopathological hallmark of PD. These neurons project their axons along the nigrostriatal pathway and release dopamine (DA) in the striatum (ST). DA deficit is a primary cause of both motor and nonmotor PD symptoms. The increased risk of dementia and mortality observed in patients arises from a complex interplay of dopaminergic and non‐dopaminergic factors as the disease progresses.^[^ ^2^, ^3^ ^]^ When early motor symptoms, such as resting tremor and bradykinesia, become recognizable, ≈50% or more of DAergic neurons in the SN have already degenerated. Given the limited regenerative capacity of the central nervous system (CNS), current treatments can only alleviate the symptoms. Traditional treatments include drug therapy,^[^ ^4^ ^]^ surgical practice, such as deep brain stimulation,^[^ ^5^ ^]^ and physical and occupational therapy.^[^ ^6^ ^]^ However, these approaches exhibit limitations, including severe adverse effects, such as dyskinesia,^[^ ^7^ ^]^ surgical complications like electrode dislocation or damage, or infections,^[^ ^8^, ^9^ ^]^ and reduced efficacy with disease progression.^[^ ^10^ ^]^ To replenish lost DAergic neurons and thereby increase local DA levels, many studies have recently begun exploring cell replacement as a potential approach to improve motor symptoms significantly.^[^ ^11^, ^12^ ^]^ Due to promising results from preclinical animal studies, clinical trials using human stem cells have been approved in China (NCT03119636)^13^, Japan (UMIN000033564, JMA‐IIA00384)^14^, the United States (NCT04802733)^15^, Australia (NCT02452723)^16^, and Europe (NCT05635409)^17^. In clinical trials, induced DAergic progenitors are transplanted directly into the ST to restore dopaminergic innervation and synaptic connections with medium spiny neurons (MSNs), thereby improving motor symptoms. Although early results indicate an improvement in motor symptoms, limitations such as low cell engraftment and survival of transplanted cells, along with incomplete differentiation in vivo,^[^ ^18^ ^]^ restrict the full potential of these approaches. In addition, with current strategies, DA neurons are transplanted into an ectopic site (ST) rather than the original site (SN) since axons must cover a long distance between these disconnected brain regions, which is impossible due to the absence of proper guiding cues in the adult brain.^[^ ^19^ ^]^ Therefore, intrastriatal grafts are currently more efficient in motor function recovery than intranigral grafts, but they result in altered brain architecture. To overcome these limitations and to reconstruct the brain, there is a great need for the exploration of innovative adjunctive strategies. A promising and novel approach for axon guidance involves the application of intracellular mechanical forces. We recently introduced a technique termed nano‐pulling, which enables remote control of cellular mechanotransduction through magnetic forces. This method involves incubating cells with magnetic nanoparticles (MNPs) at a concentration of 3.6 µg mL⁻¹ of Fe, resulting in particle uptake in both mature neurons (3–4 pg of Fe per cell)^[^ ^20^ ^]^ and neural progenitors (4–5 pg of Fe per cell).^[^ ^21^ ^]^ Once internalized, the MNPs magnetize the cells, enabling the generation of forces in the range of piconewtons upon exposure to an external magnetic field.^[^ ^20^, ^22^, ^23^ ^]^ Therefore, nano‐pulling is an approach to influence cellular biomechanics, offering great potential for remote axon guidance. Nano‐pulling has demonstrated efficacy in promoting in vitro growth of neurites in neuron‐like cells,^[^ ^23^, ^24^, ^25^, ^26^, ^27^ ^]^ and neural progenitors,^[^ ^21^ ^]^ but also mature neurons such as DAergic neurons,^[^ ^25^, ^26^ ^]^ cortical neurons,^[^ ^24^, ^26^, ^27^, ^28^ ^]^ and hippocampal neurons.^[^ ^20^, ^29^ ^]^ Remarkably, in addition to morphological changes, nano‐pulling also influences cellular functionality, promoting synaptogenesis^[^ ^20^ ^]^ and neural stem cell differentiation and maturation.^[^ ^21^, ^30^ ^]^ Recently, we demonstrated that nano‐pulling contributes to the stabilization of microtubules (MTs) in the axon that, in turn, leads to a reduction in MT turnover, resulting in their accumulation.^[^ ^29^ ^]^ At a biophysical level, this generates a contractile force that pulls the growth cone, counteracting the pulling force generated against the axon by actomyosin contraction within the growth cone, thus facilitating tip advancement.^[^ ^31^ ^]^ At the molecular level, since MTs serve as the primary cytoskeletal tracks for axonal transport, their accumulation leads to an enrichment of vesicles, including synaptic vesicles, as well as organelles.^[^ ^29^ ^]^ Interestingly, this enrichment in microtubules, endoplasmic reticulum, mitochondria, and synaptic vesicles was observed not only in mature axons but also in the projections of neural progenitors.^[^ ^21^ ^]^ This positive modulation of transport along MTs likely facilitates the addition of new mass, which is essential for axon growth and synaptic maturation of both neurons and neural progenitors under nano‐pulling.^[^ ^31^, ^32^ ^]^

While the safety of MNP‐loaded stem cells has been assessed in vivo in rats,^[^ ^33^ ^]^ further studies are needed to demonstrate their efficacy in inducing mechanotransduction, particularly in more complex models beyond in vitro cell cultures. One such model is represented by neural organotypic cultures, providing insights into stem cell behavior and their interaction with the host tissue.^[^ ^34^ ^]^ An example demonstrating the application of nano‐pulling following cell transplantation into mouse spinal cord slices revealed that the growth of neural processes can be enhanced and guided by mechanotransduction even within tissues.^[^ ^21^ ^]^ Similarly, Dhillon and colleagues tested the effect of mechanical forces on primary rat DAergic precursors transplanted in the SN of organotypic rat brain slices, observing an increase in neurite outgrowth despite no significant difference in the number of migrating cells.^[^ ^25^ ^]^ In the present study, we co‐cultured two coronal brain sections comprising areas of the SN and the ST using ventral midbrain (VM) and cortico‐striatal sections. We first systematically characterized the model at various times to identify conditions resembling the initial stages of PD pathology, a critical focus for advanced therapies. Through the axotomy of DA neurons, we induced a PD‐like state, mechanically emulating the loss of DAergic connections while preserving intact DAergic neurons. Aiming for cell transplantation, we optimized culturing conditions by evaluating the presence of neural projections and the absence of apoptosis in brain slices. Forebrain‐derived neural precursors, preloaded with MNPs, were subsequently transplanted into the SN region of the co‐cultures, followed by nano‐pulling using an external magnet. We detected increased neurite length and a change of growth direction toward the magnetic field, in addition to enhanced neuronal maturation. To confirm these effects using another clinically relevant model, we also generated induced dopaminergic (iDA) progenitor neurospheres and demonstrated that nano‐pulling promotes the outgrowth of their neural processes. These results underscore the potential of mechanotransduction in guiding the growth of neural processes of iDA progenitors transplanted within brain tissue. Furthermore, extended mechanical stimulation revealed enhanced neuronal maturation inside the brain tissue. Since both MNPs and magnetic fields are already widely used in clinical settings,^[^ ^35^ ^]^ our demonstration that mechanotransduction facilitates the ex vivo reconstruction of the natural nigrostriatal circuitry by promoting oriented neuronal growth and maturation represents a significant step toward overcoming current limitations of cell therapies without additional risks.

## 2 Results

### 2.1 Assessment of an Organotypic VM/ST Co‐culture

**Figure 1:**
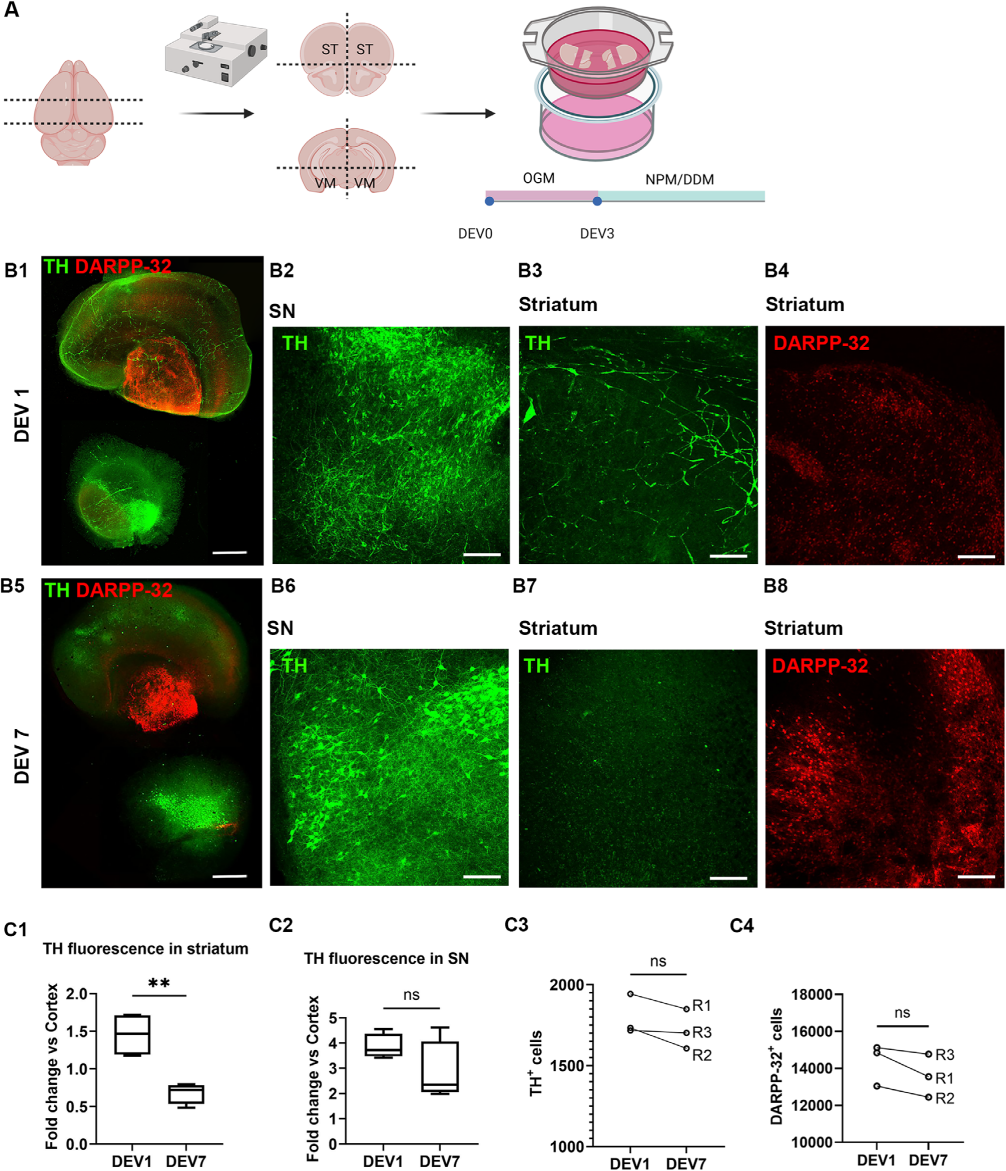
The organotypic VM/ST model. A) The mouse hemisphere is cross‐sectioned to obtain coronal dorsal striatum (ST) and ventral mesencephalon (VM), which are further cut in two halves along the horizontal plane. The upper portion of the ST slice and the lower portion of the VM slice are co‐cultured on a Millicell insert in OGM. At DEV3, OGM is replaced with the switching medium (NPM or DDM). B) TH‐ and DARPP‐32 immunostaining in VM/ST co‐culture at DEV1 (B1–4) and DEV7 (B5–8). Scale bar in B1 and B5: 700 µm. Scale bar in B2–4 and B6–8: 200 µm. C1) TH fluorescence intensity in the ST versus cortex. Bar plot (min‐to‐max). Unpaired two‐tailed *t*‐test, *p* = 0.003, *t* = 4835, df = 6, *n* = 4. C2) TH fluorescence intensity in the SN versus cortex. Bar plot (min‐to‐max). Unpaired two‐tailed *t*‐test, *p* = 0.167, *t* = 1573, df = 6, *n* = 4. C3) TH‐positive neurons per hemisphere. Wilcoxon matched‐pairs signed‐rank test, two‐tailed, *p* = 0.25, *n* = 3 (R1/R2/R3). C4) DARPP‐32‐positive neurons per hemisphere. Wilcoxon matched‐pairs signed‐rank test, two‐tailed, *p* = 0.25, *n* = 3 (R1/R2/R3).

We developed an optimized organotypic co‐culture model to address key limitations of monocultures. This model combines ventral midbrain and cortico‐striatal sections, previously shown to retain their morphological characteristics in vitro, thus allowing neurons to develop the innervation patterns of the nigrostriatal DAergic system.^[^ ^36^, ^37^ ^]^ We prepared each hemisphere by sectioning it into four dorsal ST slices and four ventral mesencephalon slices. Initially, these slices were halved along the axial (horizontal) plane, and we co‐cultured the upper portion of the ST slice with the lower portion of the VM slice on a Millicell insert (**Figure** **1**A). The VM/ST co‐cultures were maintained in a serum‐containing culture medium, previously used for organotypic neuronal slices,^[^ ^38^, ^39^ ^]^ supplemented with 100 ng mL^−1^ of brain‐derived neurotrophic factor (BDNF) (hereafter referred to as organotypic medium, OGM).

Next, we optimized the co‐culture protocol to support the survival of transplanted neurons. Since serum‐containing media interfere with neuronal differentiation, we replaced the OGM on day ex vivo (DEV) 3 with a medium that sustains the survival of both the VM/ST co‐culture and transplanted cells. Specifically, we tested a medium originally developed for differentiating neocortical neuroepithelial stem (NES) cells,^[^ ^40^, ^41^ ^]^ referred to as neural progenitor medium (NPM) and a medium proposed for the differentiation of DAergic neuron progenitors,^[^ ^42^, ^43^ ^]^ referred to as dopaminergic differentiation medium (DDM).

First, we optimized the protocol to maximize VM/ST co‐culture survival under different switching media (NPM or DDM) conditions. Previous studies have shown that reducing section size helps preserve tissue integrity and neuronal survival by improving metabolite diffusion while maintaining tissue structure.^[^ ^44^, ^45^ ^]^ Based on this, we modified section thickness (400 or 350 µm) and VM slice size, using either a half‐slice (lower portion) or a quarter‐slice (lower/external portion). In addition, we tested supplementation with insulin, BDNF, and glial cell line‐derived neurotrophic factor (GDNF), as these factors have been shown to promote neuronal survival and axonal growth in organotypic cultures.^[^ ^45^, ^46^ ^]^

Figure S1A (Supporting Information) shows VM/ST co‐cultures maintained until DEV14 under different conditions. When the co‐culture (½ ST and ¼ VM, 400 µm) was maintained in NPM, we observed a marked reduction in neurofilament (NFL) fluorescence, particularly in the lower section containing the SN, along with a significant loss of neuronal projections, which were completely absent at the VM/ST interface (Figure S1A1, Supporting Information). A loss of integrity in the midbrain section was also evident, indicating that this medium is inadequate for VM/ST co‐culture. The addition of growth factors (BDNF in Figure S1A2 (Supporting Information) or insulin+BDNF in Figure S1A3, Supporting Information) or the reduction of slice thickness (350 µm, Figure S1A4, Supporting Information) was associated with less degeneration of neural projections. However, the optimal extension of NFL‐positive neuronal projections between the VM and ST slices was achieved when co‐culturing ½ of the ST slice with ¼ of the VM slice (Figure S1A5,6, Supporting Information), especially when NPM was supplemented with BDNF and GDNF (see inset of Figure S1A6, Supporting Information). Indeed, reducing the VM slice from a half‐slice to a quarter‐slice was crucial for maintaining strong NFL expression, especially in the SN, and promoting the sprouting of neural projections in the gap region. This finding was further confirmed by experiments performed using DDM, where different VM slice sizes were compared (half‐slice in Figure S1A7, quarter‐slice in Figure S1A8, Supporting Information). The best results in terms of NFL staining were observed when the VM slice was reduced to a quarter‐slice (inset of Figure S1A8, Supporting Information). Based on this result, we selected the condition with a reduced VM size for subsequent cell transplantation experiments (i.e., condition A6 for NPM and condition A8 for DDM). A viability assay performed for all tested conditions revealed an overall low level of activated Caspase‐3 (aCasp3) staining (Figure S1B, Supporting Information).

Next, we stained VM/ST co‐cultures for neuronal markers associated with the nigrostriatal pathway, specifically, tyrosine hydroxylase (TH), to identify nigral DAergic neurons and dopamine‐ and cAMP‐regulated neuronal phosphoprotein (DARPP‐32) to label MSNs,^[^ ^47^ ^]^ which guides dopaminergic axons during the growth phase to establish the nigrostriatal connection.^[^ ^48^ ^]^

At DEV1, TH labeling was evident in both VM and dorsal ST sections (Figure 1B1). Although no TH‐positive labeling was observed in the cortex, diffuse TH labeling, likely corresponding to neuronal projections rather than cell bodies, was present in the ST section (Figure 1B3). In VM sections, TH‐positive cells were clearly visible, likely representing neurons in the SN (Figure 1B2). By DEV7, TH labeling was still abundant in the VM section (Figure 1B5,6), while, in addition to the cortical region, the striatal region also no longer showed TH‐positive labeling in either cell bodies or projections (Figure 1B7). A quantitative analysis of TH fluorescence intensity was conducted at these two times (DEV1 and DEV7) across the cortex, ST, and SN. Fluorescence intensity in the ST and SN was normalized to cortical fluorescence, which, as previously indicated,^[^ ^49^ ^]^ corresponds solely to the intrinsic autofluorescence of brain sections. In the ST, a statistically significant reduction in TH labeling was observed at DEV7 compared to DEV1 (1.46 ± 0.15 and 0.68 ± 0.07 for DEV1 and DEV7, respectively; *p* = 0.03, Figure 1C1). By contrast, no statistically significant difference was observed between the time points in the SN (3.85 ± 0.25 and 2.82 ± 0.61 for DEV1 and DEV7, respectively; *p* = 0.17, Figure 1C2). This progressive loss of TH‐positive fluorescence in the ST suggests the degeneration of DAergic axonal projections. To verify whether this result was due to the loss of connection rather than a change in the number of TH‐positive cell bodies, we performed cell counts on the five slices of VM and ST derived from the same hemisphere, ensuring that the entire SN and ST were included in the analysis. A comparison of TH‐positive cells between the two time points (Figure 1C3) revealed no significant change (1.80 ± 0.07 × 10^3^ and 1.70 ± 0.07 × 10^3^, *p* = 0.25), indicating that the midbrain tissue remained viable, with DAergic neurons surviving the tissue sectioning process. While DAergic neurons remained intact, they lost connections to their innervation targets.

At DEV1 and DEV7, DARPP‐32‐positive cells were localized in the striatal region (Figure 1B4–8), consistent with previous studies on ST organotypic cultures.^[^ ^49^, ^50^ ^]^ Diffuse DARPP‐32 positivity was also observed in VM sections, likely due to MSN projections forming synaptic connections with neurons in the SN pars reticulata, which are involved in the indirect basal ganglia pathway.^[^ ^51^ ^]^ The total count of DARPP‐32‐positive neurons in the hemispheres did not reveal any differences between the time points (1.43 ± 0.07 × 10^4^ and 1.36 ± 0.07 × 10^4^ for DEV1 and DEV7, respectively; *p* = 0.25, Figure 1C4), indicating MSNs were stably preserved in culture.

In conclusion, the VM/ST co‐culture exhibits typical features of a Parkinson's model, with progressive loss of DAergic projections but survival of MSNs and DAergic neurons, consistent with the early stages of the disease.

**Figure 2:**
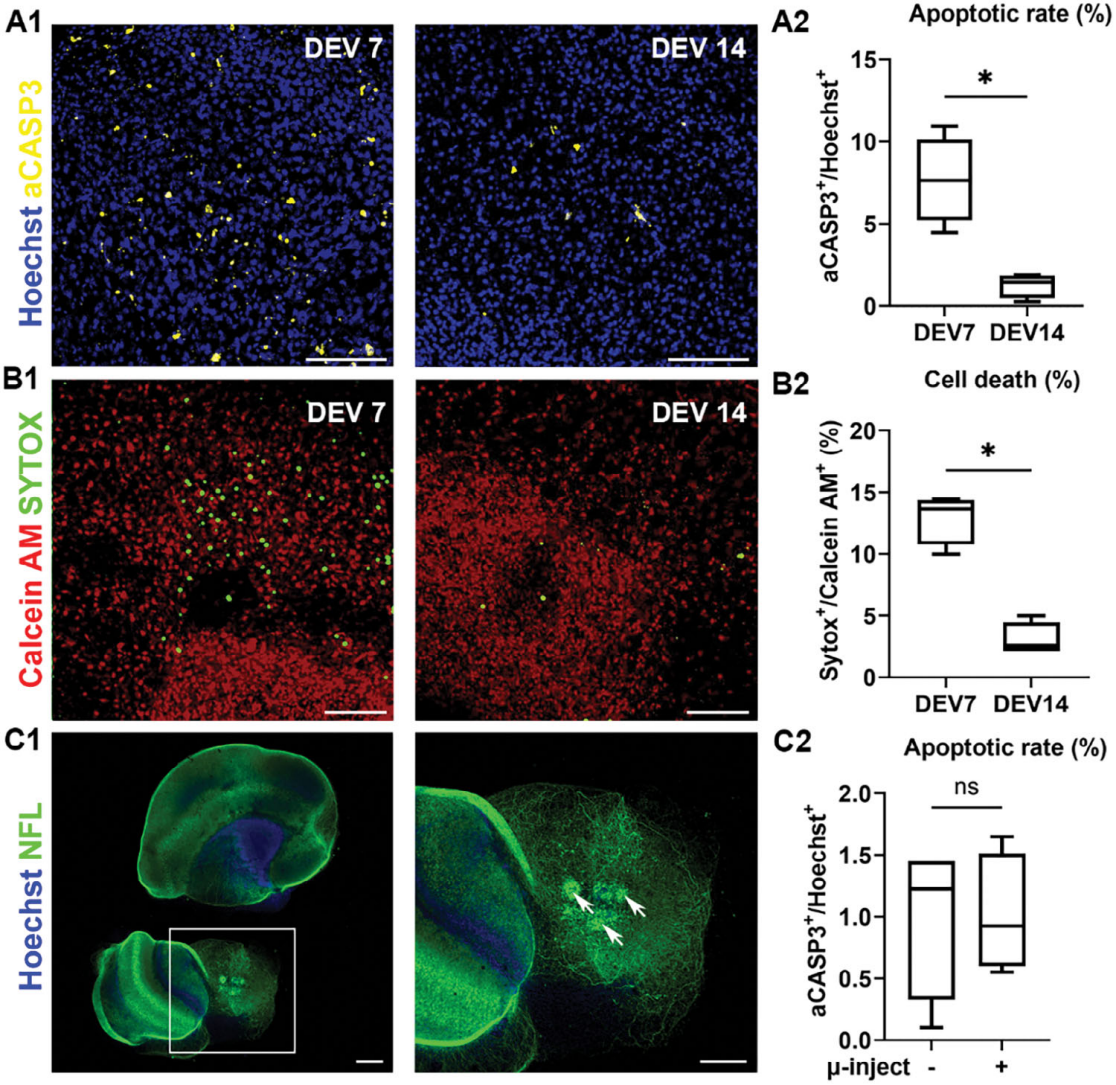
Viability assay of the VM/ST co‐culture. A1) aCASP3 (yellow) and Hoechst (blue) staining at DEV7 and 14. A2) Percentage of aCASP3^+^ cells relative to Hoechst^+^ cells. Box plot (min‐to‐max). Mann–Whitney test, two‐tailed, *U* = 0, *p* = 0.029, *n* = 4. B1) Sytox (green) and Calcein AM (red) staining at DEV7 and 14. B2) Percentage of Sytox^+^ cells relative to Calcein AM^+^ cells. Box plot (min‐to‐max). Mann–Whitney test, two‐tailed, *U* = 0, *p* = 0.029, *n* = 4. C1) VM/ST co‐culture microinjected or non‐microinjected with NES cells at DEV4. NFL (green) and Hoechst (blue) staining at DEV14. The right image is a magnification of the left image, with white arrows indicating injection sites. C2) Percentage of aCASP3^+^ cells relative to Hoechst^+^ cells at DEV14 in microinjected versus non‐microinjected conditions. Box plot (min‐to‐max). Mann–Whitney test, two‐tailed, *U* = 8, *p* = 1, *n* = 4. Scale bar: 150 µm.

Finally, we assessed cellular viability to confirm the survival of VM/ST co‐culture in the optimized protocol. The apoptotic rate, defined as the proportion of cells positive for the apoptotic marker aCASP3 relative to the total number of cells (Hoechst‐positive), decreased significantly from 7.67 ± 1.33% at DEV7 to 1.26 ± 0.37% at DEV14 (*p* = 0.0028, **Figure** **2**A1,2). Similarly, cell death, calculated as the ratio of cells positive for Sytox, which stains dead cells with compromised nuclear membrane, to cells positive for Calcein AM, a marker of viable cells, was significantly reduced from 12.94 ± 1.03% at DEV7 to 3.06 ± 0.67% at DEV14 (*p* = 0.029, Figure 2B1,2). Overall, these results suggest that after the initial cell damage caused by tissue transection, the co‐culture stabilizes in vitro. This stabilization is crucial for developing a long‐term, stable disease model suitable for subsequent cell transplantation studies. To address the potential trauma associated with microinjection for cell transplantation, we repeated the viability assay on VM/ST co‐cultures engrafted with NES cells. Normalized cell counts of aCASP3‐positive cells indicated no significant damage from the needle or injection process, with similar apoptotic rates observed in transplanted and non‐transplanted co‐cultures (1.00 ± 0.32% vs 1.01 ± 0.24%, respectively, *p* = 1, Figure 2C1,2). Notably, these results were obtained using NES cells pre‐loaded with nanoparticles, further confirming that MNP internalization does not affect the number of viable cells, in line with previous works.^[^ ^21^ ^]^

### 2.2 Nano‐Pulling Induces Guided Growth of Transplanted NES Cells

**Figure 3:**
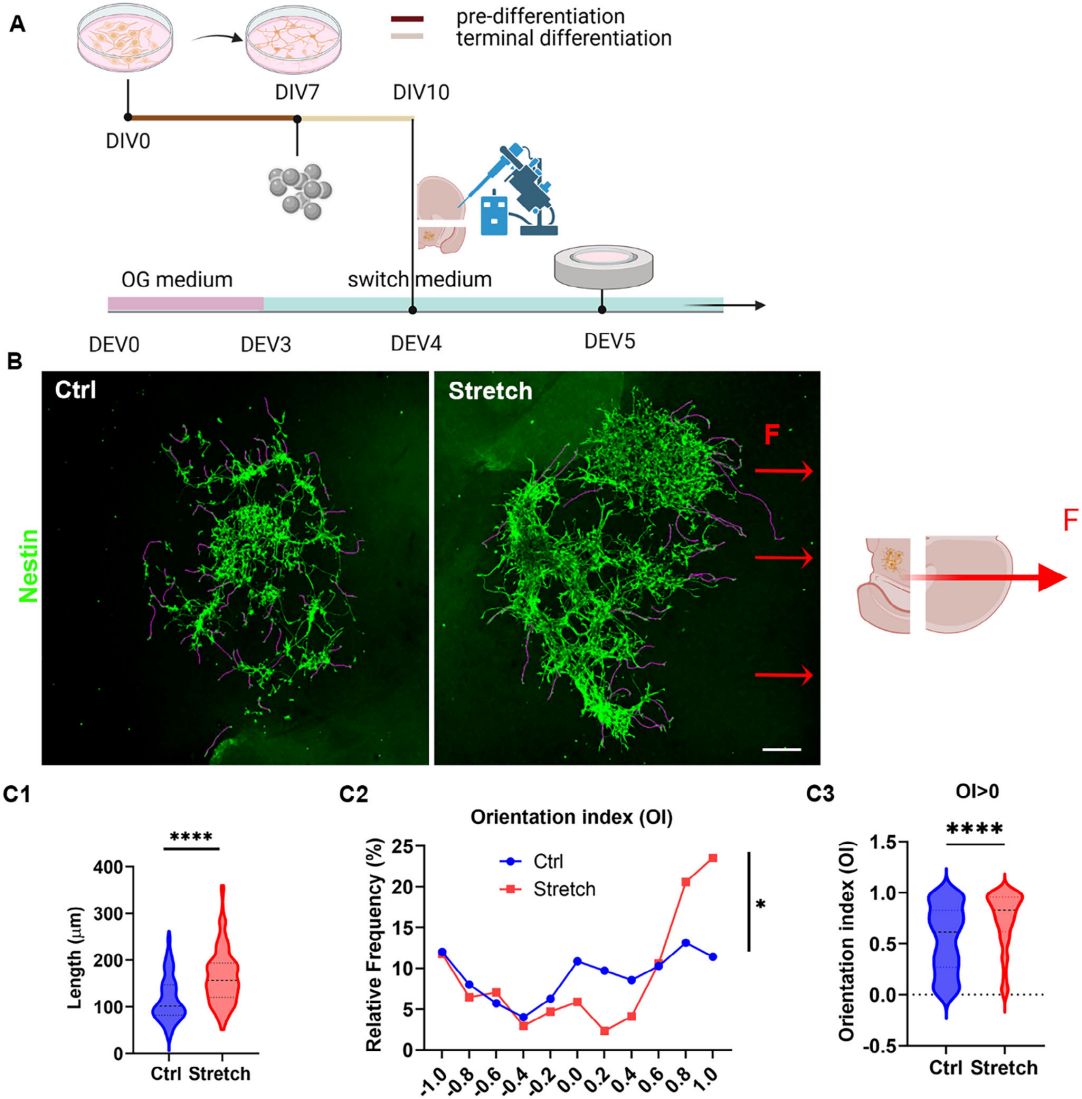
Nano‐pulling of NES cells transplanted in the VM/ST. Short‐term assay (the stretching time was 48 h). A) Timeline of the experiment. NES cells were maintained for 7 d in the pre‐differentiation medium. At day in vitro (DIV)7, cells were labeled with MNPs and incubated in the differentiation medium. At DIV10, cells were transplanted in DEV4 VM/ST. At DEV5, the stretch group was placed in the toroidal magnet. B) The neural processes of the hNestin‐positive NES cells were traced, and their length and orientation were measured. Scale bar: 200 µm. The traced neural processes are highlighted in magenta. C1) Data distribution (violin plot) of the length. Mann–Whitney test, two‐tailed, *U* = 7514, *p* < 0.0001, *n* = 168 processes. C2) Frequency of the orientation index. Unpaired two‐tailed *t*‐test, *p* = 0.039, *t* = 2.073, df = 343, n = 170 processes. C3) Data distribution (violin plot) of the OI for OI > 0. Mann–Whitney test, two‐tailed, *U* = 3882, *p* < 0.0001, *n* = 110 processes.

In a proof‐of‐concept study, human NES cells were transplanted into the VM/ST co‐culture to validate the ability of nano‐pulling to induce guided growth of their neural processes. MNP‐loaded NES cells were grafted into the VM section at DEV4 (**Figure** **3**A), and the magnetic field was applied from DEV5 to DEV7. After immunostaining, neural processes were traced using NeuronJ^[^ ^52^ ^]^ on human Nestin (hNestin)‐positive NES cells (Figure 3B). We observed a statistically significant increase in the length of processes subjected to nano‐pulling compared to the control condition (116.30 ± 3.70 and 164.00 ± 4.60 µm for the control and stretch groups, respectively; *p* < 0.0001, Figure 3C1), consistent with previous studies involving mechanical stimulation of NES cells transplanted into a spinal cord organotypic model.^[^ ^21^ ^]^ Furthermore, we verified that NES cell processes were growing inside organotypic slices (Video S1, Supporting Information).

The ability of nano‐pulling to induce guided growth was assessed by calculating the orientation index (OI), defined as the cosine of the angle α between the force vector and the growth direction of the neural process (OI = cosα). The closer the OI value is to 1 and the smaller the angle between the two vectors, the more aligned the process is with the force vector. We observed a specific increase in neuronal processes with OI close to 1 under the stretched condition (Figure 3C2), indicating that the neural processes tended to align with the force vector. Given that only force vectors directed from the soma to the tip are productive for inducing stretch‐growth,^[^ ^23^ ^]^ the analysis was restricted to the semi‐plane of −90° < α < 90°. Statistical analysis revealed a significant increase in the OI of stretched neural processes (0.80 ± 0.02, corresponding to a mean angle of 37°) compared to control processes (0.55 ± 0.03, corresponding to a mean angle of 57°) (*p* < 0.0001, Figure 3C3). Indeed, stretched processes are more aligned to the force vector than control processes, with a net difference of 20°, in line with previous results observed in in vitro neuronal cultures.^[^ ^21^, ^23^ ^]^ These findings demonstrate that nano‐pulling influences the directional growth of neural processes to preferentially align with the direction of the force vector in our PD organotypic model.

**Figure 4:**
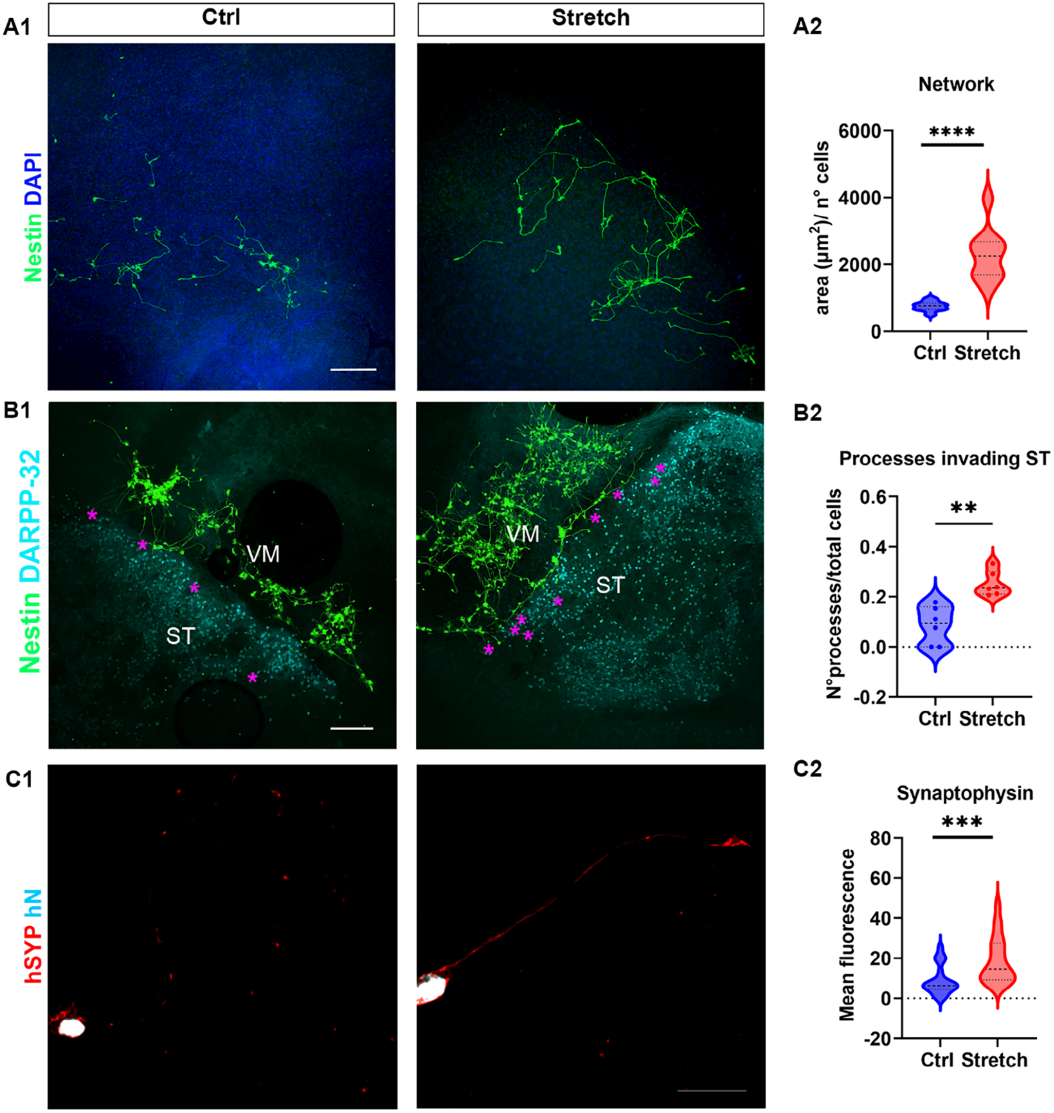
Nano‐pulling of NES cells transplanted in the VM/ST. Long‐term assay (the stretching time was 10 d). A1) Network formed by neural processes of the hNestin‐positive NES cells. Scale bar: 200 µm. A2) Data distribution (violin plot) of the network expressed as the area of the processes per unit of surface. Mann–Whitney test, two‐tailed, *U* = 0, *p* < 0.0001, *n* = 8 co‐cultures. B1) NES cells transplanted in the VM invade with their processes (hNestin‐positive) the ST (DARPP‐32‐positive). The magenta stars highlight the processes invading the ST. Scale bar: 200 µm. B2) Data distribution of the density of the processes that invade the ST (violin plot). Two‐tailed *t*‐test, *t* = 4.479, df = 10, *p* = 0.0012, *n* = 6 co‐cultures. C1) NES cells (hNuclei‐positive) transplanted in the VM express human synaptophysin (hSYP, red). Scale bar: 50 µm. C2) Data distribution of the hSYP mean fluorescence (violin plot). Mann–Whitney test, two‐tailed, *U* = 73, *p* < 0.0007, *n* = 20 cells.

In addition, we exposed NES cells transplanted into the VM/ST co‐culture to long‐term nano‐pulling (10 d) and analyzed the network of their neural processes. We measured the area of the region of interest (ROI) associated with neural processes (hNestin‐positive) and normalized it to the number of NES cells (hNuclei‐positive) (**Figure** **4**A1). We observed a twofold increase in the area (749.40 ± 47.80 and 2273.00 ± 244.20 µm^2^ for control and stretch conditions, respectively, *p* < 0.0001, Figure 4A2).

Next, we cultured the VM and ST slices without any gap by placing the two tissue pieces in direct contact with each other on the Millicell membrane to examine the ability of neural processes of cells transplanted into the VM to invade the ST region (Figure 4B1). We found a threefold increase in the number of processes sprouting into the ST region (0.09 ± 0.03 and 0.25 ± 0.02 for control and stretch conditions, respectively, *p* = 0.0012, Figure 4B2). Furthermore, we observed processes invading the ST region to grow within the tissue (Figure S2 and Video S2, Supporting Information).

Finally, we stained NES cells for human synaptophysin (hSYP), an early marker of synaptic vesicle formation^[^ ^53^ ^]^ (Figure 4C1), confirming active vesicle formation and maintained functionality. We observed a 60% increase in hSYP fluorescence (8.95 ± 1.66 and 18.78 ± 2.58 for control and stretch conditions, respectively, *p* = 0.0024, Figure 4C2).

In summary, NES cells transplanted into the VM/ST co‐culture exhibited significant sprouting of their neural processes, forming a dense network, when exposed to long‐term nano‐pulling (10 d). These processes projected from the implantation site (VM) to the ST and showed robust expression of SYP, an early marker of synaptic vesicle formation.

### 2.3 Nano‐Pulling of iDA Progenitor Neurospheres

**Figure 5:**
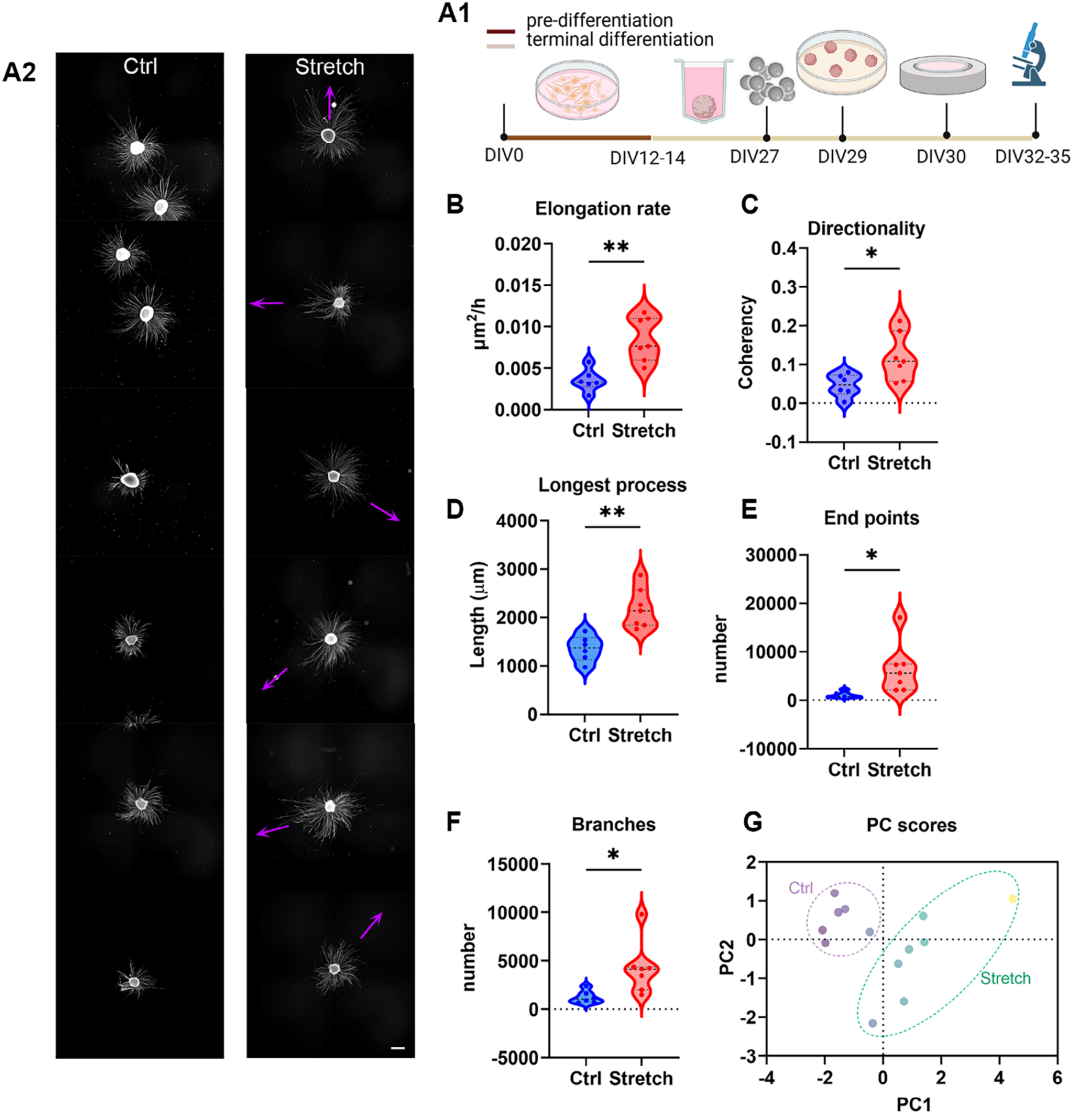
Nano‐pulling of iDA progenitor neurospheres. A1) Induced pluripotent stem cells (iPSCs) were cultivated at high density in pre‐differentiation medium until DIV12‐14. At DIV12‐14, cells were then transferred into low cell adhesion V‐shaped 96‐wells containing differentiation medium to promote neurosphere formation. MNPs were added at DIV27. Two days after MNP loading, neurospheres were transferred onto iMATRIX‐coated 35 mm dishes. The magnetic field was applied 24 h later, and the neurospheres were stimulated for 48 h. A2) iDA progenitor neurospheres cultured in control and stretch conditions (stretching time: 48 h). The magenta arrows indicate the direction of the force vector. Scale bar: 500 µm. B) Elongation rate of the neural processes projecting from the iDA progenitor neurospheres is represented as a violin plot. Two‐tailed *t*‐test, *t* = 4130, df = 11, *p* = 0.0017. C) Dominant orientation of neural projections (violin plot). Two‐tailed *t*‐test, *t* = 2640, df = 11, *p* = 0.023. D) Length of the longest projection (violin plot). Two‐tailed *t*‐test, *t* = 4220, df = 11, *p* = 0.0014. E) Number of the projections identified by endpoints (violin plot). Two‐tailed *t*‐test, *t* = 2540, df = 11, *p* = 0.0275. F) Number of branches (violin plot), two‐tailed *t*‐test, *t* = 2602, df = 11, *p* = 0.0246. G) Principal component analysis (PCA). PCs were selected to account for 88% of the total explained variance. Standardized method. *N* = 6 neurospheres iDA progenitor neurospheres for “Ctrl” and 7 neurospheres for “Stretch.”.

iDA neurospheres were incubated with MNPs overnight, which had no impact on cell viability compared to untreated neurospheres (Figure S3, Supporting Information). Subsequently, these neurospheres were transferred to iMATRIX‐coated 35 mm dishes (Figure 5A1). The neurospheres generated here were positive for the presence of TH cells (6.96 ± 2.71%, *n* = 10 neurospheres, Figure S4, Supporting Information). After incubating the neurospheres to allow attachment to the iMATRIX‐coated surface, we randomly allocated the dishes into two groups (**Figure** **5**A2). The stretch group was placed into the magnetic applicator. After 48 h, both groups were fixed and stained for tubulin to visualize their projections. Whereas neurospheres cultured in control conditions showed abundant arborization that uniformly sprouted from the neurosphere core to the periphery, marked anisotropy was observed in neurospheres subjected to nano‐pulling, with increase in the number and length of neural projections extending in the direction of the applied force (Figure 5A2I, magenta arrows point to force direction). A qualitative analysis of the branching and ramification of neural projections was performed on a semi‐plane random for control neurospheres and associated with the positive orientation of the force vector $\vec{F}$ for stretched neurospheres, as we demonstrated previously that only forces oriented from soma to tip are productive for sustaining axon growth.^[^ ^23^ ^]^

First, we calculated the elongation rate of the control group (*er_k_*) and the stretch group (*er_s_*) as follows:

(1) $$er_{k}=\frac{A\left(t\right)}{t}$$

(2) $$er_{s}=\frac{A\left(t\right)-er_{k}\cdot t_{0}}{t-t_{0}}$$

with A being the area covered by the neural projections, *t* the time in culture, and *t_0_* the time when the magnet was added. We compared the elongation rate between the control and stretched groups to determine the effect of the nano‐pulling. Through this analysis, we found a twofold increase in the elongation rate (0.0035 ± 0.0005 µm^2^ h^−1^ and 0.0085 ± 0.0010 for control and stretch conditions, respectively, *p* = 0.0017, Figure 5B), consistent with previous findings in primary neurons^[^ ^22^, ^29^ ^]^ and dorsal root ganglia^[^ ^38^ ^]^ subjected to nano‐pulling. When stretched, the neural projections exhibited a preferential orientation, as indicated by the coherency index, which increased proportionally with the presence of a dominant orientation^[^ ^49^ ^]^ (0.046 ± 0.012 and 0.118 ± 0.023 for control and stretch conditions, respectively, *p* = 0.0023, Figure 5C). Furthermore, nano‐pulling increased the maximum length that a projection could reach; the length of the longest projection sprouting from the neurosphere was 1.36 ± 0.11 × 10^3^ and 2.19 ± 0.16 × 10^3^ µm for control and stretch conditions, respectively, (*p* = 0.0014, Figure 5D). The analysis of the skeletonized processes was used to verify the effect of stimulation on branching. We found a sixfold increase in the number of skeletonized projection terminal ends (1051 ± 252 and 6490 ± 1958 for control and stretched neurospheres, respectively, *p* = 0.0275, Figure 5E). A similar increase was observed in detected branches (1250 ± 265 and 4219 ± 1025 for control and stretched neurospheres, respectively, *p* = 0.025, Figure 5F). Finally, we analyzed multiple parameters (elongation rate, orientation, max length, number of branches, and number of endpoints) collectively using principal component analysis (PCA) to highlight biological differences. PCA distinctly segregated the neurospheres into two clusters, indicating that neurospheres subjected to nano‐pulling formed a coherent population, clearly distinguishable from controls (Figure 5G, PC1 and PC2 account for 88% of the total variance).

**Figure 6:**
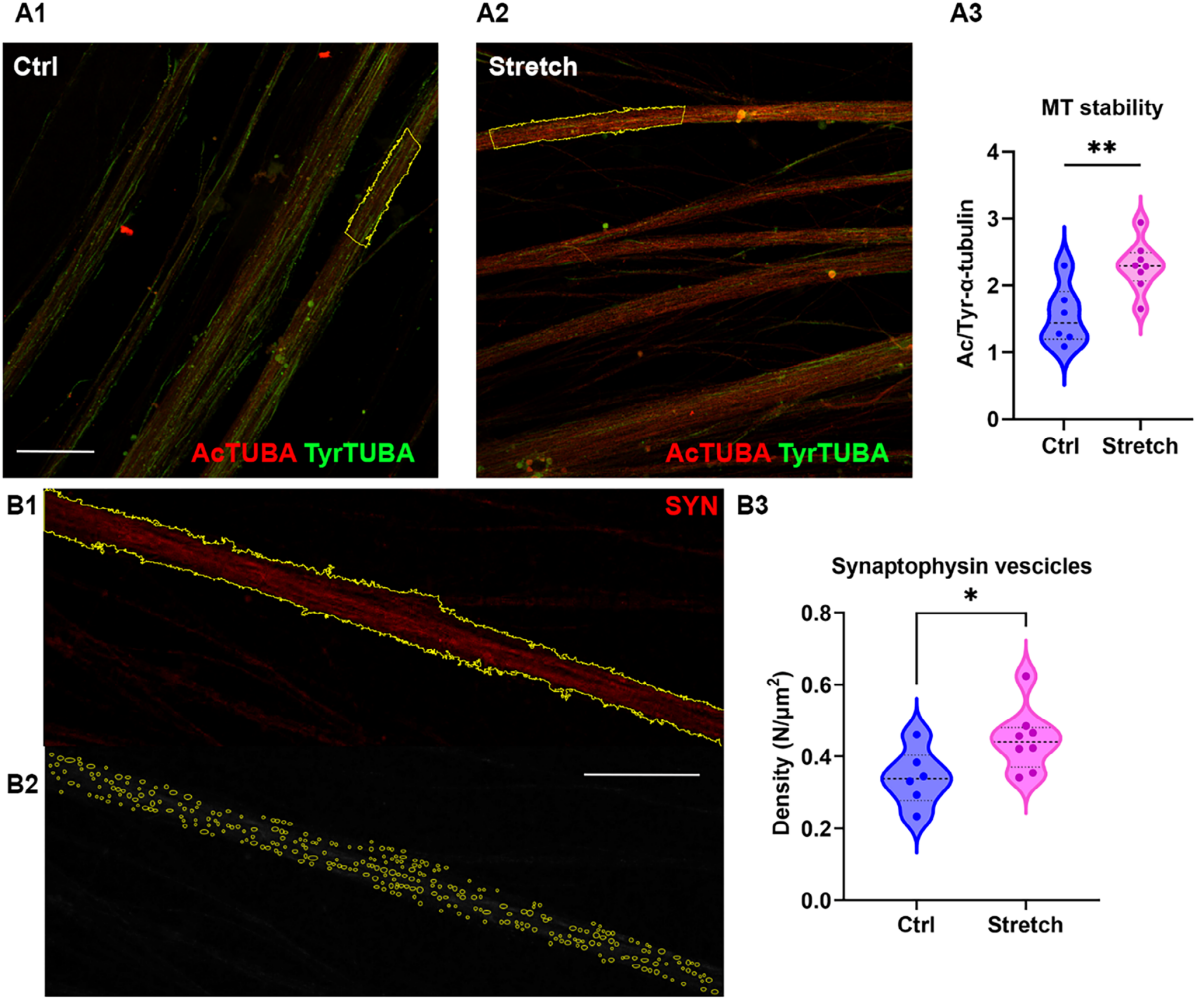
MT stability and synaptophysin vesicles in iDA progenitor neurospheres. The experimental timeline follows Figure 5A1 with an extended stretching duration of 5 d. A) MT stability: representative images for control (A1) and stretch (A2) groups. Scale bar: 50 µm. Anti‐acetylated α‐tubulin (red), anti‐tyrosinated α‐tubulin (green). The yellow contours indicate examples of ROI. A3) Quantification of the acetylated‐to‐tyrosinated α‐tubulin fluorescence ratio. Violin plot. *N* > 6 neurospheres. Unpaired *t*‐test, *p* = 0.0054, *t* = 2.111, df = 22. B) Synaptophysin (SYN) vesicle tracking. B1) ROI of an axon bundle (yellow outline). B2) SYN vesicles (yellow circles) automatically identified within the ROI. Scale bar: 50 µm. B3) Quantification of SYN vesicle density. Violin plot. Mann–Whitney test, *U* = 8, *p* < 0.043, *n* > 6 neurospheres.

Recently, we proposed that nano‐pulling promotes MT stabilization.^[^ ^29^ ^]^ Given that α‐tubulin acetylation is linked to stable MTs, while tyrosination is associated with dynamic instability,^[^ ^54^ ^]^ we performed immunostaining to examine these two post‐translational modifications of α‐tubulin. Our analysis of axon bundles in neurospheres revealed a stronger α‐tubulin acetylation signal in the stretch group (red, **Figure** **6**A2), while α‐tubulin tyrosination was more prominent in the control group (green, Figure 6A1). The ratio between α‐tubulin acetylation and tyrosination levels is widely used as an indicator of MT stability,^[^ ^54^ ^]^ and has been extensively validated in our previous studies.^[^ ^29^, ^55^ ^]^ Quantification of this ratio in axon bundles (yellow ROI, Figure 6A1,2) revealed a significant increase following nano‐pulling (1.55 ± 0.18 in control and 2.29 ± 0.13 in stretch group, *p* = 0.0054, Figure 6A3), indicating enhanced MT stability in stretched neurospheres.

We recently reported that nano‐pulling also stimulates synaptic maturation, with pre‐synaptic vesicles serving as an early marker.^[^ ^21^, ^29^ ^]^ In this study, we estimated the density of large vesicles positive for synaptophysin in axon bundles projecting from the neurospheres (Figure 6B1,2). After five days of nano‐pulling, vesicle density increased significantly (0.34 ± 0.03 vs 0.45 ± 0.03 vesicles µm^−2^ in control versus stretch condition, *p* = 0.043, Figure 6B3), in line with previously reported results for NES cells subjected to nano‐pulling during terminal differentiation.^[^ ^21^ ^]^

## 3 Conclusion

Restoring the brain's original architecture is highly desirable for next‐generation cell replacement therapies in the CNS. In the context of PD, this involves the critical task of reconstructing the nigrostriatal pathway. Here, we demonstrate that nano‐pulling is a promising tool in promoting and guiding axon growth. To evaluate this novel approach, we developed and optimized a co‐culture model of the SN and the ST, which reassembles the early stages of PD, that can serve as a platform to study the guided reconstruction of the nigrostriatal pathway. We employed this model to showcase the capacity of nano‐pulling to enhance neurite elongation, alignment, and synaptic maturation of human cortical neuronal progenitors, thus facilitating a partial reconnection between the SN and the ST in this model. In addition, we validated that nano‐pulling is applicable for clinically relevant cells by testing its application with human iDA progenitor neurospheres. Indeed, we successfully achieved guided neurite growth, resulting in increased neurite length and branching, thereby reinforcing the versatility and potential therapeutic applications of this technique.

Our results present a foundation for addressing limitations in current regenerative approaches, including insufficient axon guidance and synaptic integration. This approach possesses realistic translational potential as the safety and efficacy of clinical‐grade magnetic nanoparticles have been extensively tested.^[^ ^56^ ^]^ However, particle characteristics (size, shape, surface charge, coating) significantly influence the biological and immunological effects of MNPs, and further optimization of these properties could enhance their effectiveness and safety, supporting their clinical translation.^[^ ^57^ ^]^ Our findings indicate that intracellular iron levels progressively decrease after 2–3 weeks (Figure S5, Supporting Information), supporting the notion that MNPs undergo controlled degradation and clearance. Nanoparticles degrade and release iron ions, which are then processed through physiological iron metabolism pathways.^[^ ^58^, ^59^ ^]^ While this degradation process facilitates MNP clearance, the fate of the released iron is a critical factor influencing biocompatibility. Some studies suggest that excessive iron release may lead to oxidative stress and cytotoxicity,^[^ ^60^, ^61^, ^62^ ^]^ particularly when MNP concentrations exceed the cell's iron‐buffering capacity or when the nanoparticles are ultrasmall.^[^ ^63^ ^]^ However, evidence supports that under well‐controlled physicochemical conditions—specifically with appropriate dose, coating, and size—MNPs can be efficiently metabolized without leading to long‐term intracellular persistence or unwanted accumulation.^[^ ^64^, ^65^ ^]^ Furthermore, as nanoparticles are only applied to a relatively small population of cells before transplantation, the total iron load is low compared to systemic applications of nanoparticles contrast agents for magnetic resonance imaging.^[^ ^66^ ^]^ Nonetheless, achieving optimal conditions in complex biological environments, especially in vivo, remains challenging and requires tailored design and fine‐tuning of nanoparticle properties. Developing MNPs with controlled degradation profiles would ensure efficient clearance once their mechanotransduction role is fulfilled, thus preventing prolonged intracellular persistence. Indeed, while further optimization in the degradation kinetics and surface characteristics is needed to support clinical translation, the favorable safety and efficacy demonstrated by iron oxide MNPs in clinical applications provide a strong foundation for continued development and repurposing.^[^ ^67^, ^68^, ^69^, ^70^, ^71^, ^72^ ^]^ Beyond conventional surface functionalization, strategies for selective delivery to specific neuronal populations or subcellular domains may improve uptake, limit off‐target effects, and enhance integration into host tissue. Moreover, understanding how different neural cell types respond to MNPs—particularly in vivo—will be essential to ensure safety and reproducibility across various therapeutic contexts. Future studies should focus not only on the long‐term effects of nano‐pulling ex vivo, but also on integration and functional outcomes in vivo, including synaptic connectivity and behavior, to advance nano‐pulling as a therapeutic strategy for repairing damages caused by neurodegenerative diseases.

Our work bridges the fields of nanomaterials and cell transplantation, thereby paving the way for innovative therapies that leverage remote manipulation of mechanical forces to reconstruct neural networks and restore natural functional connectivity in the CNS.

## 4 Experimental Section

The raw data, metadata, and processed data that support the findings of this study are openly available in ZENODO repository at https://doi.org/10.5281/zenodo.14604511.

### Maintenance of hiPSCs

Human iPSCs (1231A3) were maintained in StemFlex medium (#A3349401, Gibco; Thermo Fisher Scientific, Waltham, MA) on laminin‐511 fragment‐coated (0.5 µg cm^−2^, iMatrix‐511 silk; #NP892‐021; Matrixome Corp., Osaka, Japan) culture plates. After 7 d, cells were washed with DPBS and detached with 0.5× CTS TrypLE Select Enzyme (#A1285901; Gibco; Thermo Fisher Scientific, Waltham, MA) in DPBS. After centrifugation, cells were resuspended in StemFlex medium containing Y‐27632 (10 × 10^−6^ m; #030‐24021; FUJIFILM Wako Pure Chemical Co., Ltd., Japan). iPSCs were seeded at a density of 1.3 × 10^4^ cells per iMATRIX‐coated six‐well. The medium was changed every other day.

### Differentiation of hiPSCs to Dopaminergic Progenitor Cells

hiPSCs were differentiated according to a modified version of the previously published protocol.^[^ ^33^ ^]^ In short, wells of a six‐well plate were coated with 1.0 µg cm^−2^ iMATRIX in DPBS overnight at 37 °C and 5% CO_2_. iPSCs were detached as described above. The cell pellet was resuspended in 8% GMK medium (GMEM, # 11710035, Gibco, Thermo Fisher Scientific, Waltham, MA; 8% KSR, #10828028, Gibco, Thermo Fisher Scientific, Waltham, MA; 1% sodium pyruvate, # S8636, Sigma‐Aldrich, St. Louis, MO; 1% 2‐mercaptoethanol, #198‐15781, FUJIFILM Wako Pure Chemical Co., Ltd., Japan; 1% non‐essential amino acids, #11140050, Gibco, Thermo Fisher Scientific, Waltham, MA) and cells were counted. 5 × 10^6^ cells/6‐well were transferred in 4 mL 8% GMK medium supplemented with Y‐27632 (10 × 10^−6^ m), LDN193189 (100 × 10^−9^ m, # 04‐0074, Stemolecule, Reprocell, Kanagawa, Japan), and A83‐01 (500 × 10^−9^ m, #2939, Tocris, Bristol, UK). On day 1, cells were washed with DPBS and 4 mL 8% GMK medium supplemented with LDN193189 (100 × 10^−9^ m), A83‐01 (500 × 10^−9^ _m_ ), FGF8 (100 ng mL^−1^), and purmorphamine (2 × 10^−6^ m). On the second day, the supernatant was removed, and 4 mL of the same medium was added. On day 3–6, the supernatant was removed daily, and 6 mL 8% GMK medium supplemented with LDN193189 (100 × 10^−9^ m), A83‐01 (500 × 10^−9^ m), FGF8 (100 ng mL^−1^, # 069‐04373, FUJIFILM Wako Pure Chemical Co., Ltd., Japan), purmorphamine (2 × 10^−6^ m, # 166‐23991, FUJIFILM Wako Pure Chemical Co., Ltd., Japan), and CHIR99021 (3 × 10^−6^ m, # 04‐0004‐10, Stemolecule, Reprocell, Kanagawa, Japan) was added. On day 7–11, the supernatant was removed, and 8 mL 8%GMK medium with LDN193189 (100 × 10^−9^ m) and CHIR99021 (3 × 10^−6^ m) was added. On day 12, the same medium was used but supplemented with Y‐27632 (30 × 10^−6^ m) to support survival during subsequent procedures. On day 13, cells were detached with 0.5× TrypLE Select Enzyme in DPBS. After centrifugation, cells were resuspended in NB‐27 medium (Neurobasal Medium, #21103049, Gibco, Thermo Fisher Scientific, Waltham, MA; B‐27 Supplement (50×), minus vitamin A, #12587010, Gibco, Thermo Fisher Scientific, Waltham, MA; 1% penicillin‐streptomycin, #15070063, Gibco, Thermo Fisher Scientific, Waltham, MA). For each well of a low cell adhesion V‐shaped 96 well (#MS‐9096VZ, Sumitomo Bakelite Co., Ltd., Tokyo, Japan), 3 × 10^4^ cells in 150 µL of DDM composed of NB27 supplemented with Y‐27632 (30 × 10^−6^ m), GDNF (10 ng mL^−1^, #212‐GD, R&D Systems, Minneapolis, MN), ascorbic acid (200 × 10^−6^ m, A15613.0E; Thermo Fisher Scientific, Waltham, MA; #A4034, Sigma‐Aldrich, St. Louis, MO), BDNF (20 ng mL^−1^, #248‐BD, R&D Systems, Minneapolis, MN), and dbcAMP (400 × 10^−6^ m, #D0627, Sigma‐Aldrich, St. Louis, MO) were added. These low cell adhesion V‐shaped 96 wells support the rapid settling and neurosphere formation of the cells, which is necessary for the successful differentiation into DAergic progenitors. A partial medium change was performed every third day by aspirating 70 µL and adding 80 µL fresh medium (without Y‐27632) per 96 well. During the differentiation, cells were incubated at 37 °C and 5% CO_2_.

### Ethical Statements

All research involving NES cells adhered to the NIH guidelines for obtaining and distributing human tissue for biomedical research, with approval granted by the Human Investigation Committees and Institutional Ethics Committees of the respective institutes providing the samples. The University of Pisa's Committee on Bioethics also granted final approval (Review No. 29/2020). Human specimens, de‐identified for privacy, were supplied through the Joint MRC/Wellcome Trust grant (099175/Z/12/Z) from the Human Developmental Biology Resource (www.hdbr.org). Proper informed consent was obtained, and non‐identifying information for each specimen was duly recorded. Tissue handling followed ethical guidelines and regulations for research use of human brain tissue, as outlined by the NIH (http://bioethics.od.nih.gov/humantissue.html) and the WMA Declaration of Helsinki (http://www.wma.net/en/30publications/10policies/b3/index.html).

Animal procedures strictly adhered to protocols approved by the Italian Ministry of Public Health and the local Ethical Committee of the University of Pisa, in accordance with Directive 2010/63/EU (Project License No. 39E1C.N.5Q7 granted on 30/10/2021). C57BL/6J mice were maintained in a controlled environment (23 ± 1 °C, 50 ± 5% humidity) with a 12‐h light‐dark cycle, and had ad libitum access to food and water.

### Maintenance and Differentiation of Human NES Cells

Human NES cells were previously derived from the neocortex, as described in prior reports.^[^ ^36^ ^]^ For the maintenance of NES cells, a medium composed of Dulbecco's minimum essential medium/F12 (DMEM/F12; #11330‐032; Gibco; Thermo Fisher Scientific, Waltham, MA) supplemented with B27 (1:1000, #175040‐44; Gibco; Thermo Fisher Scientific, Waltham, MA), N2 (1:100, #17502‐048; Gibco; Thermo Fisher Scientific, Waltham, MA), 20 ng mL^−1^ fibroblast growth factor 2 (FGF2; #13256029; Gibco; Thermo Fisher Scientific, Waltham, MA), 20 ng mL^−1^ epidermal growth factor (EGF; #PHG0311; Gibco; Thermo Fisher Scientific, Waltham, MA), 1.6 mg mL^−1^ glucose, 20 µg mL^−1^ insulin (#I9278, Sigma, St. Louis, MO), and 5 ng mL^−1^ BDNF (#PHC7074; Gibco; Thermo Fisher Scientific, Waltham, MA) was used. Cells were maintained in T12.5 flasks pretreated with POLFN, composed of 0.01% poly‐l‐ornithine (#P4957; Sigma, St. Louis, MO) supplemented with 5 µg mL^−1^ laminin (#23017‐015, Invitrogen, Waltham, MA) and 1 µg mL^−1^ fibronectin (#354008, Corning, Corning, NY). POLFN solution was left at 37 °C for 1 h. Subsequently, three washes with cell culture‐grade water were performed. Every 2–3 d, half of the culture medium was replaced with fresh medium. Cells were passaged approximately every 5 d upon confluence. After washing with Dulbecco's phosphate‐buffered saline (DPBS; #14190‐094; Gibco; Thermo Fisher Scientific, Waltham, MA), cells were detached by incubation with 0.25% trypsin with EDTA (Gibco; Thermo Fisher Scientific, Waltham, MA). After adding a trypsin‐inactivating solution, i.e., 10% fetal bovine serum (FBS; #A5256701; Gibco; Thermo Fisher Scientific, Waltham, MA) in DPBS, the suspension was centrifuged for 5 min at 200×*g*. After removing the supernatant, cells were resuspended in a maintenance medium and plated at a density of ≈0.5–1 × 10^5^ cells cm^−2^. Finally, the ROCK inhibitor Y‐27632 (10 × 10^−6^ m; #SCM075; Sigma, St. Louis, MO) was added to support cell survival. NES cells underwent neuronal differentiation in two stages. For the pre‐differentiation phase, cells were seeded in NES medium without FGF‐2 and EGF for 7 d, with medium changes every 2–3 d. In the terminal differentiation phase, cells were dissociated and replated at a density of 0.8–1 × 10^5^ cells cm^−2^ in a medium composed of DMEM/F12 (1:2), neurobasal (1:2; #21103‐049; Gibco; Thermo Fisher Scientific, Waltham, MA), N2 (1:200), B27 (1:100), insulin (10 µg mL^−1^), l‐glutamine (1:100; #35050‐038, Gibco; Thermo Fisher Scientific, Waltham, MA), and BDNF (30 ng mL^−1^). During terminal differentiation, at DIV10, cells were detached and used for transplantation.

### Organotypic Co‐Culture of Ventral Midbrain and Cortico‐Striatal Sections

For organotypic co‐cultures, P4 pups were used. For dissection and isolation, a protocol proposed by Croft and Noble was modified.^[^ ^47^ ^]^ Briefly, the brain was isolated within a cold dissection medium containing DMEM/F12 and 6.5 mg mL^−1^ glucose. The brain was posteriorly cut to remove the brainstem, cerebellum, and thalamus using a scalpel blade. Subsequently, the remaining part was incised along the interhemispheric fissure to obtain two separate hemispheres. These were then transferred to a new dish containing cold and fresh dissection medium. The hemispheres were kept on ice for 10–15 min. Meanwhile, culture insert membranes with pore size 0.4 µm (#PICM0RC50; Millicell; Sigma, St. Louis, MO), pre‐coated with collagen (100 µg mL^−1^; #C7661; Sigma, St. Louis, MO), poly‐l‐lysine (100 µg mL^−1^; #P4707; Sigma, St. Louis, MO), and laminin (10 µg mL^−1^; #L2020; Sigma, St. Louis, MO), were washed three times with culture medium. The OGM is composed of minimum essential medium (MEM; #11090‐081; Gibco; Thermo Fisher Scientific, Waltham, MA) with 25% of horse serum (#16050‐122; Gibco; Thermo Fisher Scientific, Waltham, MA), 25 × 10^−3^ m N‐2‐hydroxyethylpiperazine‐N‐2‐ethane sulfonic acid (HEPES; #15630‐056; Gibco; Thermo Fisher Scientific, Waltham, MA), 25% (vol/vol) Hanks’ Balanced Salt Solution (HBSS; #14025‐050; Gibco; Thermo Fisher Scientific, Waltham, MA), 6.5 mg mL^−1^ glucose, 2 × 10^−3^ m l‐glutamine, 100 U mL^−1^ penicillin, and 100 µg mL^−1^ streptomycin. For brain slice generation, a strip of filter paper (#1001‐240, Whatman, Sigma, St. Louis, MO) was placed on the cutting platform of a tissue chopper (#TC752, McIlwain Tissue Chopper, Campden Instruments, England). After adding a few drops of dissection medium, half of the brain was placed on the filter paper and oriented to obtain coronal sections with 350 or 400 µm thickness. The same procedure was repeated for both hemispheres. The sections containing the SN and the dorsal ST were carefully separated and cut with a scalpel blade to obtain the portions for the co‐culture and transferred to culture insert membranes. Up to five co‐cultures consisting of the upper portion of the dorsal ST section and the lower portion of the ventral midbrain section were placed on inserts. If not specified otherwise, a gap of 1 mm between the two portions was measured under an optical microscope to ensure experimental reproducibility. In addition, each co‐culture was placed with a radial orientation, with the portion corresponding to the dorsal ST 1.4 mm close to the edge of the membrane insert. After 30 min of incubation at 37 °C, the medium was replaced with fresh medium, supplemented with 100 ng mL^−1^ BDNF (#SRP3014; Sigma, St. Louis, MO). Co‐cultures were kept in humified conditions at 37 °C with 5% CO_2_. The medium was changed completely on DEV1 to maintain co‐cultures. From DEV3, the medium was replaced every other day with DDM or NPM composed of Neurobasal, N2 (1:200), B27 (1:100), l‐glutamine (1:100), 6.5 mg mL^−1^ glucose, 100 ng mL^−1^ BDNF, and 100 ng mL^−1^ GDNF (#SRP3200; Sigma, St. Louis, MO).

### Magnetic Nanoparticles and Magnetization

For the internalization into NES cells, Viscover iron oxide magnetic nanoparticles were used (XL FeraSpin; #130095173; Viscover, Berlin, Germany) with a hydrodynamic diameter of 50–60 nm. Four hours after plating for terminal differentiation, Viscover nanoparticles corresponding to a Fe concentration of 100 × 10^−6^ m were added directly to the medium. For the internalization into DA progenitors, Chemicell iron oxide magnetic nanoparticles (#4115; Chemicell, Berlin, Germany) with a hydrodynamic diameter of 100 nm were used. 5 µg mL^−1^ Chemicell nanoparticles (corresponding to 65 × 10−6 m Fe) in neurosphere medium were added to the 96‐wells one day before transferring. The intracellular iron was quantified with an iron assay kit (#DIFE‐250, BioAssay Systems, Hayward, California), and the absorbance was measured at a wavelength of 590 nm. Figure S5 (Supporting Information) provides information on MNP internalization, expressed as an increase in intracellular Fe levels.

### Cell Transplantation

NES cells at an early stage of differentiation (DIV10) were grafted into the ventral mesencephalon of co‐culture slices at DEV 4. For transplantation, NES cells were labeled in suspension with the CellTrace Cell Proliferation Kit (#C34567, Invitrogen, Thermo Fisher Scientific, Waltham, MA), following the provider's instructions. In particular, cells were incubated with the marker for 20 min at 37 °C and again for 5 min after adding an inactivating solution with 10% FBS in DPBS. At the end of this process, the cell suspension was centrifuged for 5 min at 200 *g*. Then, cells were resuspended in fresh differentiation medium with the addition of 10 × 10^−6^ m Y‐27632 to achieve a concentration of ≈35000 cells µL^−1^. The cell suspension was loaded into a glass microneedle, and the injection volume was calibrated, corresponding to ≈150 cells. On DEV4, cells were transplanted at three injection sites at the ventral mesencephalon level of each co‐cultured slice. Depending on the experimental requirements, cells were transplanted into co‐cultures with or without the gap. This approach was designed to prevent cellular processes from halting at the boundary or otherwise being impeded upon reaching the edge of the transplanted section. The successful injection of a sufficient number of cells was verified using a fluorescence microscope by observing a red fluorescent signal derived from the CellTrace labeling.

### Magnetic Field and Nano‐Pulling

To exert the mechanical force required for nano‐pulling, magnetic applicators suitable for holding 35 mm Petri dishes were used. The magnetic field, with a constant intensity of 46.5 T m^−1^ and a radial centrifugal direction, was generated by eight magnets positioned within the device with a toroidal configuration.^[^ ^22^ ^]^ Figure S6A (Supporting Information) illustrates the magnetic field distribution within the applicator, while Figure S6B1,2 (Supporting Information) shows the flux density (B) and its gradient (dB/dx) along the radial direction, highlighting the region where images were acquired in the Petri dishes. A schematic representation of a Petri dish inside the applicator, with arrows indicating the radial outward force, is provided in Figure S6C (Supporting Information).

For the nano‐pulling of DAergic neuron progenitors, neurospheres were washed in warm DPBS and transferred to plastic Petri dishes. To support neurosphere attachment, 750 µL of DDM supplemented with 1.0 µg cm^−2^ iMATRIX was added. Three hours later, an additional 750 µL of DDM was carefully added. The day after transferring, the dish with neurospheres subjected to nano‐pulling was randomly chosen and placed inside the magnetic device (stretch group). The control condition was kept outside the magnetic applicator (ctrl group).

For NES cells, the day after the transplantation, Petri dishes used for nano‐pulling were placed in the magnetic devices (stretch group), while the control Petri dishes were kept without the magnetic applicators (ctrl group). The duration of magnetic field application depended on the specific experiment. At the end of this period, co‐cultures underwent fixation and immunostaining.

### Immunostaining

Organotypic co‐cultures were washed with DPBS on the undersides of the membrane and subsequently fixed with 4% formaldehyde (FA) for 30 min: the first 15 min with FA only beneath the membrane and the remaining 15 min with the solution above. After fixation, three DPBS washes were performed on both sides of the membrane. The membrane was separated from the plastic ring using a scalpel to conduct the immunostaining free‐floating. Co‐cultures were permeabilized with 0.7% Triton X‐100 in DPBS for 10 min. Subsequently, a blocking solution consisting of 0.5% Triton X‐100 and 10% FBS in DPBS was added and maintained for 4 h at 4 °C. The solution was replaced with an antibody solution consisting of 0.5% Triton X‐100, 1% FBS in DPBS, and primary antibodies. The antibody solution was maintained overnight at 4 °C. The dilutions for primary antibodies were as follows: NFL (1:500; #MAB1615; Sigma, St. Louis, MO), NFL (1:500; #AB2565456; Biolegend, San Diego, CA), Caspase 3 (aCASP3) (1:500; #9661; Cell Signaling, Danvers, MA), human Nestin (hNestin) (1:500; #MAB1259; R&D, Minneapolis, MN); TH (1:300; #22941; Immunostar, Hudson, WI); TH (1:350; #HPA061003; Millipore, Burlington, USA), DARPP‐32 (1:500; #MAB1259; R&D, Minneapolis, MN), human Nuclei (hNuclei) (1:400; #MAB1281; Sigma, St. Louis, MO), hSYP (1:500; #50‐6525‐82; Invitrogen, Thermo Fisher Scientific, Waltham, MA). The next day, three DPBS washes were performed, and the second antibody solution, including secondary antibodies (1:500; #AB2633280; #AB2633277; #AB2534088; #AB143157; Thermo Fisher Scientific, Waltham, MA) and Hoechst33342 (1:1000; #H3570; Invitrogen, Thermo Fisher Scientific, Waltham, MA), was added and protected from light for 3 h at room temperature (RT). After three 3‐min washes with DPBS, membranes were mounted on glass slides. For this, a drop of Aqua‐Poly/Mount solution (#18606‐20; Aqua‐Poly/Mount, Polysciences) was placed on the surface of a glass slide, with membranes laid on top. Another drop of Aqua‐Poly/Mount was placed on a coverslip, which was slid over the co‐cultures. Slides were allowed to dry in the chemical hood overnight and subsequently imaged.

Two partial washes with DPBS were performed to prevent detaching neurospheres from the dish before fixation with 4% FA for 10 min. Following three 3‐min washes with DPBS‐Triton X‐100 (PBSX) (0.1% Triton X‐100 in DPBS Ca^2+^/Mg^2+^), samples were permeabilized using a permeabilization solution (0.5% Triton X‐100 in DPBS Ca^2+^/Mg^2+^) and blocked at RT for 1 h with a blocking solution (5% fetal bovine serum, 0.3% Triton X‐100 in DPBS Ca^2+^/Mg^2+^). Primary antibodies specific for β‐tubulin III (TUBB3) (1:500; #T8578; Sigma, St. Louis, MO), TH (1:750; #HPA061003; Millipore, Burlington, USA), tyrosinated α‐tubulin (1:400; #ab6160; Abcam, Cambridge, UK), acetylated α‐tubulin (1:400; #T7451; Sigma‐Aldrich, St. Louis, MO), SYP (1:500, #Mab329, Millipore, Burlington, MA) were incubated for 2 h at RT in the antibody solution (3% fetal bovine serum, 0.2% Triton X‐100 in DPBS Ca^2+/^Mg^2+^). Samples were then washed three times for 3 min and incubated for 1 h at RT with secondary antibodies (1:500, #AB143157; #AB2534088; Life Technologies, Carlsbad, CA) and Hoechst 33342 (1:1000; #H3570; Invitrogen, Thermo Fisher Scientific, Waltham, MA). Fixed cells were directly imaged after two 3‐min washes with PBSX and one 3‐min wash with DPBS Ca^2+/^Mg^2+^.

Images of NES cells and organotypic co‐cultures were acquired using a laser scanning confocal microscope (Nikon, Eclipse Ti). Images of neurospheres were acquired using a fluorescence microscope (Nikon, TE2000‐U).

### Vitality and Cytotoxicity Assay

The LIVE/DEAD Viability/Cytotoxicity Assay Kit (#L32250, Invitrogen, Thermo Fisher Scientific, Waltham, MA) was used to assess the vitality of organotypic co‐cultures. The kit includes Sytox (1:2000) and Calcein AM (1:2000), which were added to the culture solution, both beneath and above the membrane. In particular, the two components were directly added to the fresh culture medium beneath the membrane, and a drop of the working solution was applied to each section of co‐cultures above the membrane. The staining solution was incubated for 30 min at RT and protected from light. The membrane was separated from the plastic ring using a scalpel and placed upside down on a glass coverslip. Finally, a few drops of DPBS were added above the membrane to prevent drying before and during image acquisition. Live images were acquired by a confocal microscope as quicky as possible.

To assess the vitality assay of the iDA neurospheres, 5 µg mL^−1^ Chemicell nanoparticles were added to each well on day 28 of differentiation, as described in the section “Magnetic nanoparticles and magnetization”. After 24 h of incubation, ten neurospheres were collected and washed twice with DPBS. Neurospheres were dissociated with Neuron Dissociation Solution S (#297‐78101, FUJIFILM Wako Pure Chemical Co., Ltd., Japan) according to the manufacturer's instructions to obtain single cells. Single cells were stained with Acridine Orange/Propidium Iodide Stain (F23001, logos biosystems, Gyeonggi‐do, South Korea) and analyzed by a fluorescent cell counter (LUNA‐FL, L20001, logos biosystems, Gyeonggi‐do, South Korea) with the following parameters (size gating 6–30 µm, green fluorescence threshold/exposure: 8/6, red fluorescence threshold/exposure: 8/6).

### Image Analysis

The ImageJ software^[^ ^73^ ^]^ was used to examine cell viability in vitro and ex vivo by quantifying the fluorescence level and the presence of specific markers in slices, as well as measuring the elongation, orientation, and fluorescence of transplanted cells in the organotypic slices and neurospheres.

Cell viability and mortality were analyzed manually using the “Cell Counter” command on 20× magnification pictures acquired with a Z‐spacing of 2 µm. To assess cell viability, the markers aCASP3, which detects the active form of Caspase 3, and Hoechst were used. The apoptotic rate was calculated as the ratio of cells positive for the aCASP3 marker to the total number of cells positive for Hoechst and expressed as a percentage. A minimum of 1000 cells was counted for each replicate. For the ex vivo analysis, it was necessary to identify three stacks in the central region of slices before starting the count. The same approach was followed to determine the mortality rate. Live and dead cell counting was performed manually using the “Cell Counter” command of the ImageJ software. Here, 10x magnification pictures were acquired with a Z‐spacing of 2 µm. Both the live/dead and the aCASP3/Hoechst quantifications were performed as previously described in ref. [74].

For the TH expression analysis in the three regions of interest (cortex, ST, and SN), stacks of 10× magnification images were merged using the “Z project” command. The image was then converted to 8 bits and duplicated. The mean fluorescence in the cortex and ST was measured using the “Freehand Selection” tool followed by the “Measure” command, which was set to calculate the mean gray value. For the SN, the calculation of the mean gray value followed different steps, as this region contains somas and processes of TH‐positive neurons. A copy of the SN image was used to delimit the ROI to areas with only TH‐positive DAergic neurons. To achieve this, after the threshold setting, the image was converted to binary, the “Watershed” command was applied, and the ROIs were selected using the “Analyze Particles” command. The ROIs, referring to TH‐positive particles, were saved and loaded into the original 8‐bit converted image. Finally, the mean gray value of ROIs was calculated. For each of the three regions in the co‐culture, mean fluorescence values were determined at three different areas of the region, and the mean fluorescence of the region was calculated as the mean of these values. The expression of tyrosine hydroxylase in the ST and SN was quantified as the fold change in mean fluorescence compared to the cortex. Indeed, as shown in Daviaud et al.,^[^ ^49^ ^]^ in organotypic slices, TH cortex fluorescence corresponds solely to the intrinsic autofluorescence of the sections and can thus be used for normalizations.

To assess the number of cells expressing TH in the ST and exhibiting positive staining for DARPP‐32 (also known as PPP1R1B), images captured at 10× magnification with a Z‐stack of 2 µm were used. Stacks were merged using the “Z‐project” command for both quantifications. After executing the “Threshold” command, images were converted to binary format and then subjected to selection using the “analyze particles” command after the “watershed” application. The number of ROIs obtained was used to quantify the number of cells positive for both markers.

The Fiji plugin NeuronJ^[^ ^52^ ^]^ was used to evaluate the elongation of the processes of transplanted cells, the methodology described in Figure S6 in ref. [21]. Briefly, hNestin‐positive processes were blindly traced from 20× magnification images with a Z‐spacing of 2 µm. All the non‐interconnected processes above a cut‐off of 20 µm were measured. To examine the directionality of processes, NeuronJ was used to trace the hNestin‐positive, non‐interconnected processes. Subsequently, the initiation and endpoints of each tracing were used to measure the direction of individual processes. The angle α between the direction of each process and the applied force was measured. Axonal orientation was quantified as the OI, defined as OI = cos α, as described in refs. [21, 23].

For the network evaluation, the “Watershed” selection was used, followed by the “Analyze particle” command, with segments for inclusion or exclusion verified manually. ROIs were saved, with the sum of values considered as the area. Then, values were divided by the number of cells counted manually with the “Cell Counter” command. The analysis was performed with 10× magnification images acquired at the central plane, as described in detail in ref. [21].

To evaluate the number of processes invading the VM, DARPP‐32 staining was used to delineate the border between the two slices. Then, a region with 100 µm depth from the border was considered (Figure S7, Supporting Information), and the number of cells within this region was manually counted with the “Cell Counter” command. Then, the number of hNestin‐positive processes that reached the DARPP‐32‐positive region was counted. The number of processes was then normalized by the total number of cells in the region considered.

For synaptophysin evaluation, 60× images with a Z‐spacing of 0.5 µm were acquired and analyzed blindly. After 8‐bit conversion, the mean fluorescence was measured with the image background subtracted. In particular, hNestin staining was used to trace the ROI of each cell, and then the somatic region was excluded. Three different regions were selected to calculate the mean background value.

For in vitro neurosphere morphology, longest elongation process, and direction analyses, 4× magnification images were used. The elongation rate was determined using a previously described method.^[^ ^55^ ^]^ For the control condition, the analyzed semi‐plane was chosen randomly, while in the stretched samples, the semi‐plane corresponding to the positive orientation of the force vector was used. For the analysis of the longest process extending from the neurosphere, a straight line was drawn from the border of the neurosphere to the tip of the longest process in both conditions, and its length was measured. Measures of directionality were obtained using the semi‐automated Fiji plugin OrientationJ, with default settings.^[^ ^75^ ^]^ To quantify the number of endpoints and branches images were converted to binary, and the command “Skeletonize” followed by the “Analyze Skeleton” plugin was used after excluding the ROI of the neurosphere. Here, the number of endpoints represents the count of free ends in the skeletonized structure, while the number of branches corresponds to the total number of distinct segments in the skeletonized image.

To assess the number of cells expressing TH in neurospheres as a result of differentiation, images captured at 80× magnification with a Z‐stack of 1 µm were used. TH, the rate‐limiting enzyme in dopamine production, was used as a marker. The TH‐positive rate was calculated as the ratio of TH‐positive and total DAPI‐positive cells, expressed as a percentage. A total of nearly 2500 cells were counted. It was necessary to identify approximately five to eight stacks in the central region of the neurospheres before starting the count. TH‐positive cell counts were performed manually using the “Cell Counter” command in the ImageJ software.

Microtubule stability was evaluated by calculating the ratio of acetylated to tyrosinated α‐tubulin mean fluorescence, as described in ref. [55]. Random ROIs were selected on axon bundles (examples of ROI shown in Figure 6A).

The density of presynaptic vesicles was determined by analyzing SYN‐positive projections. Random ROIs were selected on axon bundles (examples of ROI shown in Figure 6B). The Fiji plugin ComDet was used to detect large vesicles with a size of one pixel (≈280 nm), with the intensity threshold for synaptophysin signal of 3 (in standard deviations) obtained by the signal‐to‐noise ratio as the mean dot intensity divided by the mean baseline background intensity.^[^ ^76^ ^]^

### Statistical Analysis

The data were plotted and analyzed using GraphPad software, version 9.0.0.121. Values were reported as the mean ± standard error of the mean (SEM). Outliers have been removed by using the ROUT method. The normality of data distribution was assessed using the Kolmogorov–Smirnov normality test. The statistical significance of viability counts was determined using the chi‐square test. The statistical significance of numerical variables was assessed using the Mann–Whitney test for non‐normally distributed data and the Student's *t*‐test for normally distributed data. For all presented analyses, significance was set at *p* ≤ 0.05.

## Conflict of Interest

The authors declare no conflict of interest.

## Supporting information



Supporting Information

Supplemental Video 1

Supplemental Video 2

## Data Availability

The data that support the findings of this study are openly available in ZENODO at https://doi.org/10.5281/zenodo.14604511, reference number 14604511.
