# Supplementary material for: Mechanical Forces Guide Axon Growth through the Nigrostriatal Pathway in an Organotypic Model
Source: Adv Sci (Weinh). 2025 May 11;12(31):2500400. doi: 10.1002/advs.202500400 (PMC12376662; doi:10.1002/advs.202500400)
Supplement: Supplementary file 1 — Supporting Information [file ADVS-12-2500400-s002.docx]

Supporting Information

Title: Mechanical forces guide axon growth through the nigrostriatal pathway in an organotypic model

Sara De Vincentiis, Elena Capitanini, Karen Kira, Claudia Dell’Amico, Jun Takahashi, Marco Onorati, Fabian Raudzus*, Vittoria Raffa*


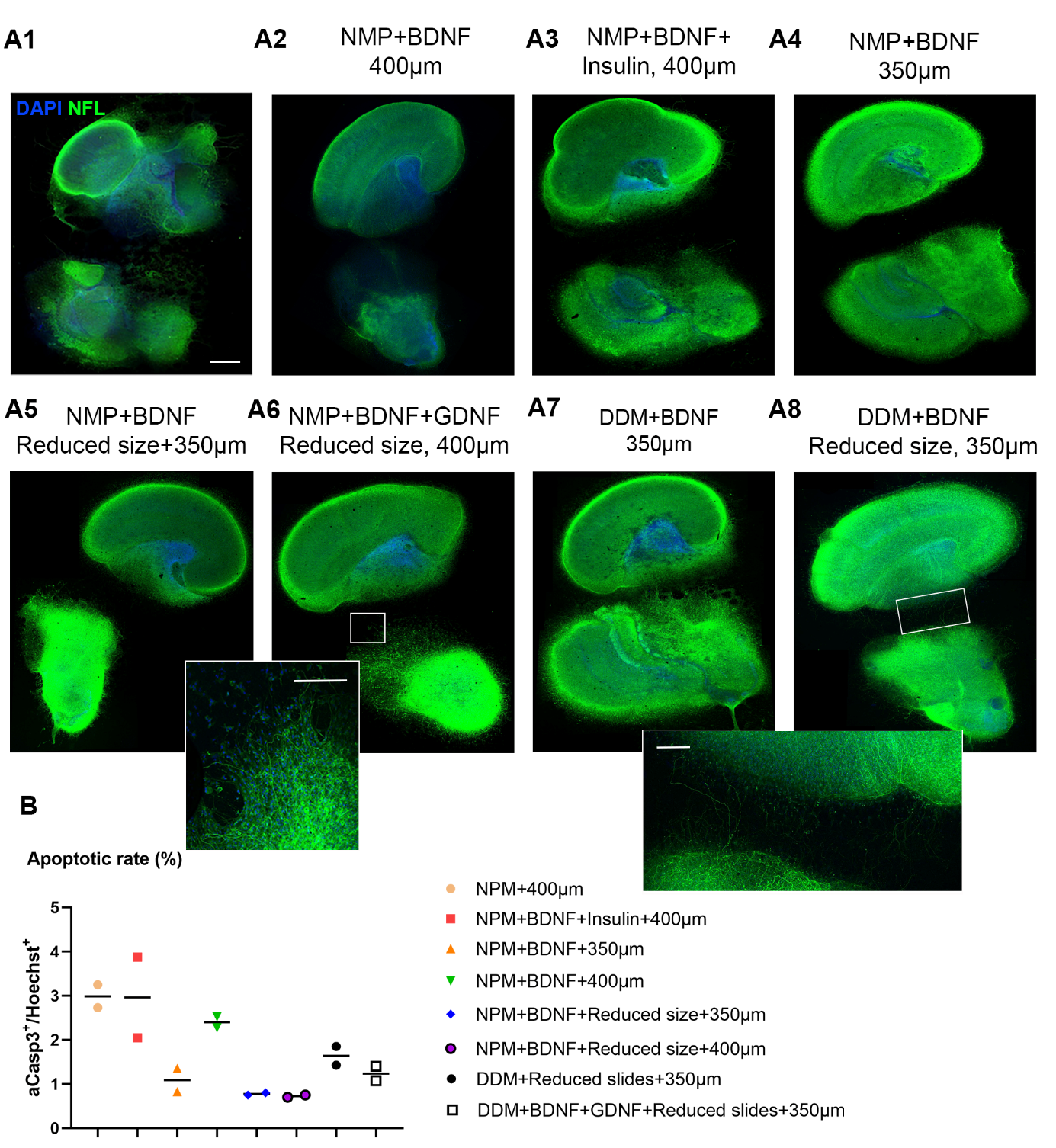
Figure S1. Optimization of VM/ST co-culture conditions: impact of section thickness, size, and neurotrophic factors. A) Representative immunofluorescence images of VM/ST co-cultures under different tested conditions. Co-cultures were maintained with different section thicknesses (400 µm vs. 350 µm) and sizes (reduced VM refers to a quarter of slice), in different media compositions (NMP and DDM) with or without growth factors. Neurofilament staining (NFL, green) highlights neuronal processes, while DAPI (blue) marks cell nuclei. A1) The co-culture (400 µm, ½ ST and ½ VM) maintained in NMP shows neuronal degeneration in both ST and VM. A3) The co-culture (400 µm, ½ ST and ½ VM) maintained in NMP supplemented with BDNF and insulin, and A4) the co-culture (350 µm, ½ ST and ½ VM) maintained in NMP supplemented with BDNF, both show low NFL fluorescence, particularly in the VM, and lack of projections in the gap. A5) The co-culture (350 µm, ½ ST and ¼ VM) maintained in NMP supplemented with BDNF shows very high NFL fluorescence in the VM but no projections in the gap. A6) The co-culture (350 µm, ½ ST and ¼ VM) maintained in NMP supplemented with BDNF and GDNF show very high NFL fluorescence and projections extending into the gap (see inset). A7) The co-culture maintained in DDM (350 µm, ½ ST and ½ VM) supplemented with BDNF, shows limited projections in the gap. A8) The co-culture in DDM (350 µm, ½ ST and ¼ VM) supplemented with BDNF, shows high neurite sprouting, including into the gap (see inset). Scale bars: 700 μm (A1-A8) and 300 μm (insets). B) Viability of VM/ST co-cultures under different conditions. Percentage of aCASP3-positive cells relative to Hoechst-positive cells. N=2. Individual values and mean reported.


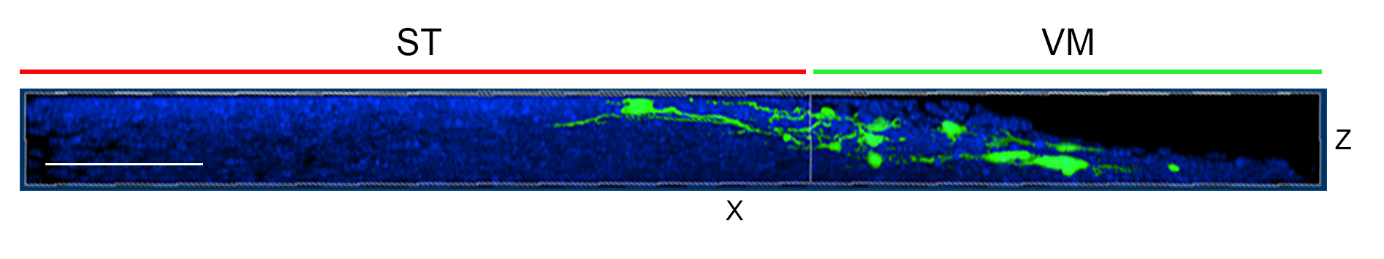


**Figure S2.** XZ-plane view of a 3D reconstruction showing NES cell processes 14 days after engraftment into VM sections. The striatum (ST) and the ventral midbrain (VM) regions are indicated by red and green lines, respectively. Anti-hNestin staining (green) highlights the NES cells and their processes, while DAPI staining (blue) marks nuclei in the tissue. The spatial organization confirms that NES cell processes extend within the organotypic section. Scale bar 100 µm.


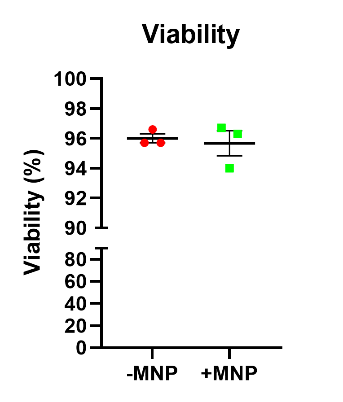




Figure S3. Viability of neurospheres after 24 h co-incubation with Chemicell nanoparticles. Neurospheres were prepared as described in 4.2. Data represents the results, mean, and SEM of three technical replicates, each containing 10 pooled neurospheres from the same preparation. Neurospheres exposed to 5 μg/ml Chemicell nanoparticles for 24 h showed no difference in viability compared to untreated neurospheres. 2-tailed t-test, t=0.3732, df=4, p=0.7279.


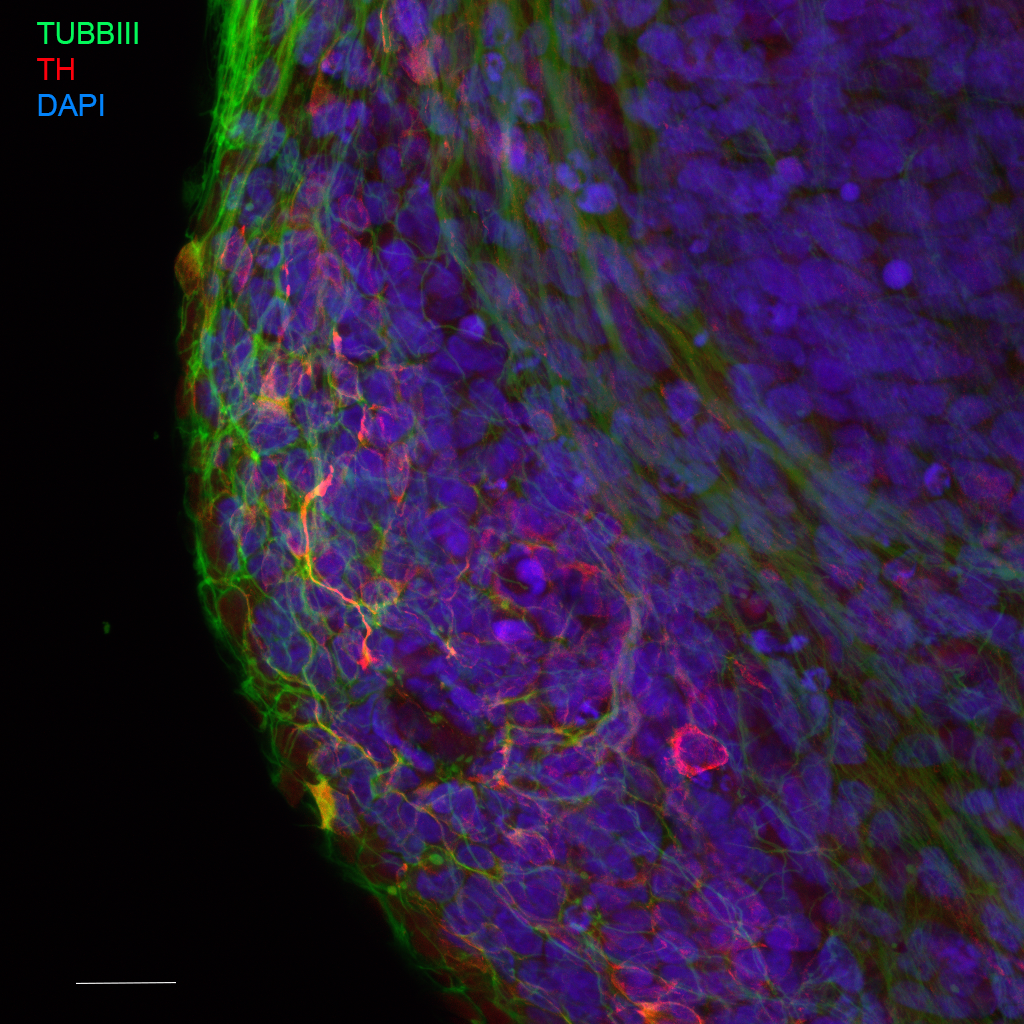


**Figure S4.** Image of a neurosphere. Anti-TUBBIII (green), anti-TH (red), DAPI (blue). Scale bar: 100 μm.


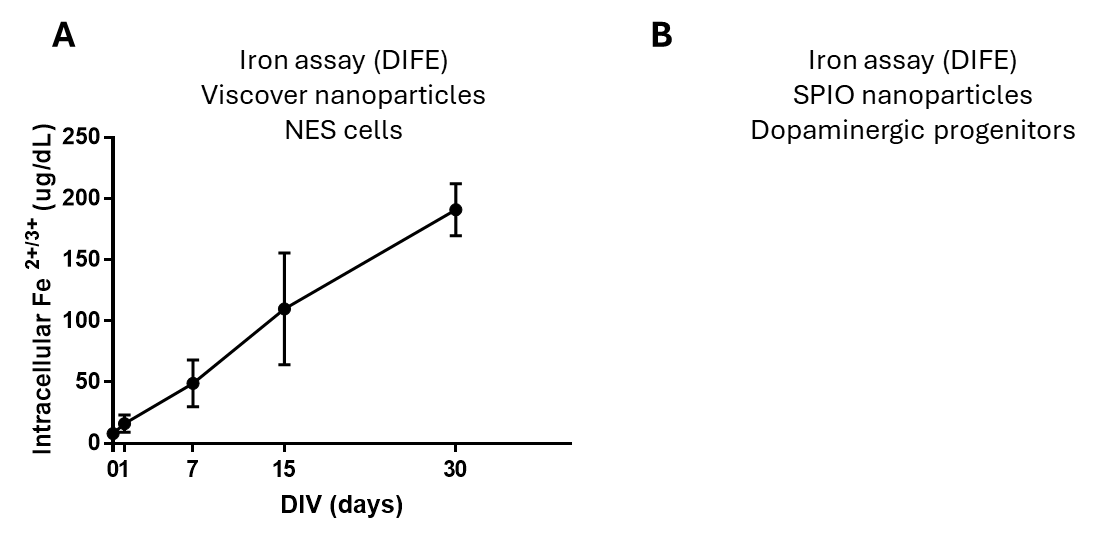


**Figure S5**. Intracellular iron quantification in NES cells. Intracellular level of Fe^2+/3+^/cell at different time points after Viscover^TM^ addition at time point 0. N=3 replicates, Mean ± SEM.


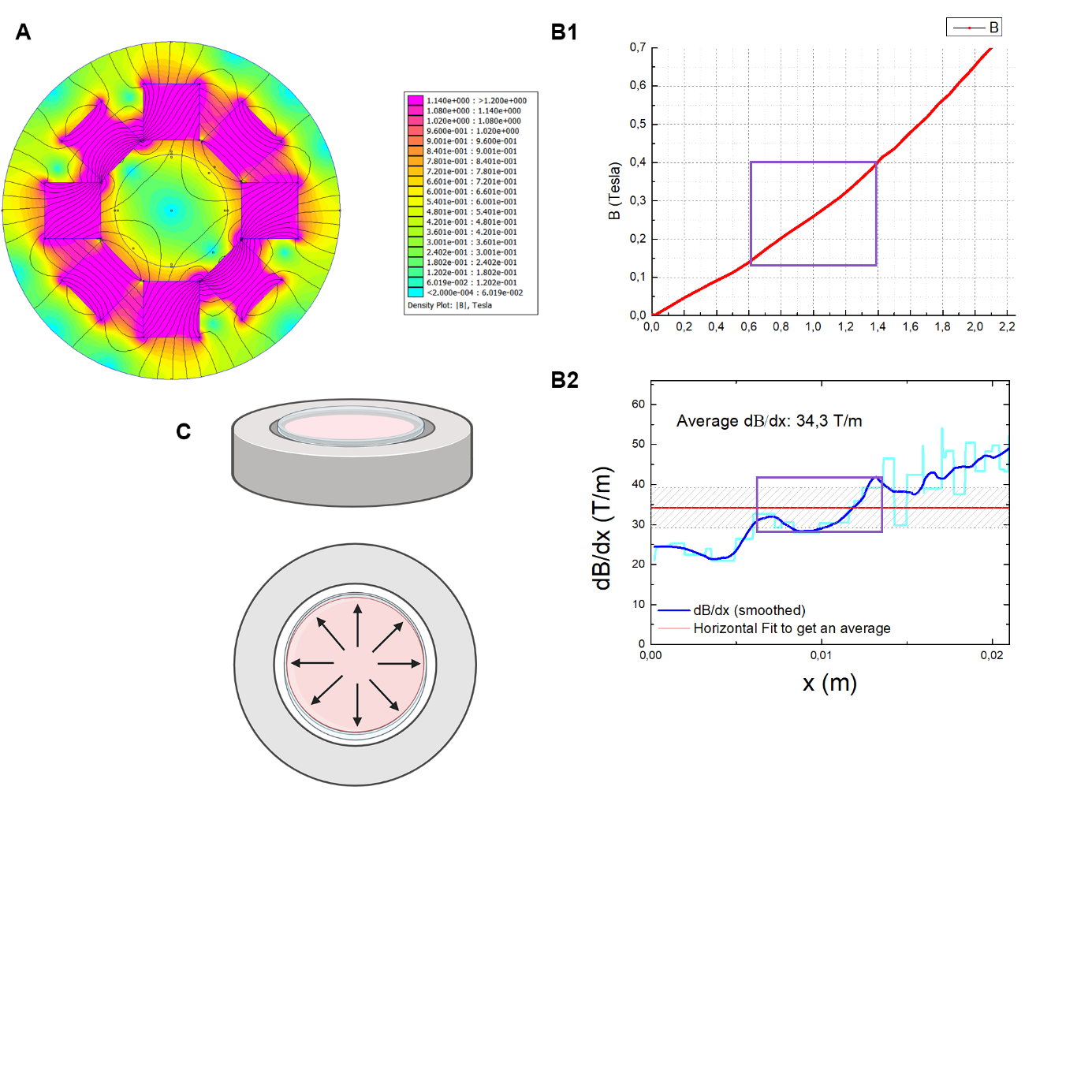


**Figure S6.** The Magnetic Applicator. A) Finite Element Method (FEM) modelling of the magnetic field inside the applicator. B1) Flux density (B) distribution within the magnetic applicator. B2) Flux density gradient (dB/dx) along the radial direction inside the applicator. The magenta rectangle highlights the region where images were taken in the Petri dishes. C) Schematic representation of a Petri dish inside the magnetic applicator. Arrows indicate the radial outward force. Modified from ^[17]^.


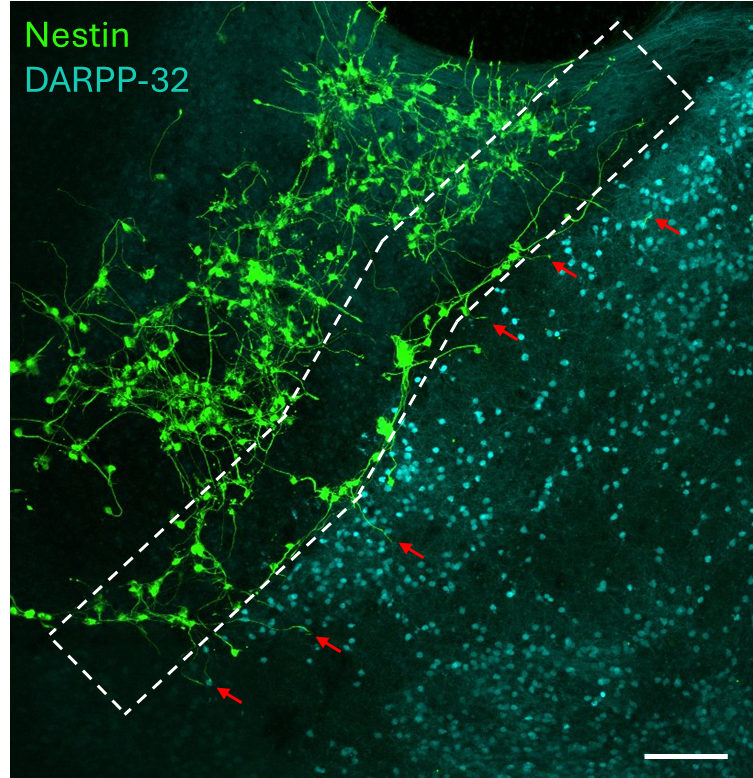


**Figure S7.** Representative image of the analysis performed on NES cells (green) transplanted in the VM/ST and reaching the DARPP-32 positive region (cyan). The dashed box highlights the area 100 μm away from the border where cells considered for the analysis are located. Red arrows indicate processes reaching the ST. Scale bar: 100 μm.

**Video S1.** 3D reconstruction of the Z-stack of NES cells four days after engraftment into VM sections. Anti-hNestin staining (green) highlights the transplanted NES cells and their processes extending into the surrounding tissue, while DAPI staining (blue) marks the nuclei of cells within the organotypic section, providing a reference for the tissue structure. Scale bars: 100 μm.

**Video S2.** 3D reconstruction of the Z-stack of NES cell processes 14 days after engraftment into VM sections. Anti-hNestin staining (green) marks NES cells and their extending processes, while DAPI staining (blue) highlights the nuclei of resident cells, outlining the organotypic tissue structure. Arrows indicate processes extending from the VM into the SN. The depth information from the Z-stack confirms that these processes are growing within the tissue rather than along its surface. Scale bars: 100 μm.
